# Supplementary material for: The master energy homeostasis regulator PGC-1α exhibits an mRNA nuclear export function
Source: Nat Commun. 2023 Sep 7;14:5496. doi: 10.1038/s41467-023-41304-8 (PMC10485026; doi:10.1038/s41467-023-41304-8)
Supplement: Supplementary file 1 — Supplementary Information [file 41467_2023_41304_MOESM1_ESM.pdf]

The master energy homeostasis regulator PGC-1α exhibits a novel mRNA nuclear export function

Simeon R. Mihaylov[1,2,10], Lydia M. Castelli[1,10], Ya-Hui Lin[1,10], Aytac Gül[1], Nikita Soni[1], Christopher Hastings[1], Helen R. Flynn[3], Oana Păun[4], Mark J. Dickman[5,6], Ambrosius P. Snijders[3,7], Robert Goldstone[8], Oliver Bandmann[1,6,9], Tatyana A. Shelkovnikova[1,6], Heather Mortiboys[1,6,9], Sila K. Ultanir[2] and Guillaume M. Hautbergue[1,6,9,*]

* Correspondence: g.hautbergue@sheffield.ac.uk (G.M.H.)

This Supplementary Information file contains:

- Supplementary Methods 1-8
- Supplementary Figures 1-10
- Supplementary Tables 1-3
- Supplementary Note 1
- Supplementary References

Supplementary Method 1 | Recombinant protein expression and purification of GB1-tagged PGC-1α domains.

All PGC-1α domains were cloned into pET24b-GB1-6xHis vector and transformed into *E. coli* BL21-RP cells. The expression of recombinant PGC-1α proteins was induced for 3 h at 37°C with 0.4 mM isopropyl-β-D-thiogalactoside (IPTG, Calbiochem) in Terrific Broth and purified by metal ion affinity chromatography (IMAC) on TALON/Cobalt beads (Clontech) using 1M NaCl containing buffers to prevent the potential co-purification of *E. coli* RNA (Lysis buffer: 50 mM Tris-HCl pH 8.0, 1M NaCl, 0.5% Triton X-100; Wash buffer: 50 mM Tris-HCl pH8.0, 1M NaCl, 0.5% Triton X-100, 5 mM imidazole). Elution was achieved with the wash buffer containing 200 mM imidazole (50 mM Tris-HCl pH8.0, 500 mM NaCl, 200 mM imidazole). Domain 1 and CBM of PGC-1α were soluble, however, the rest of the domain proteins were insoluble and present in inclusion bodies in the bacterial pellets (Supplementary Fig. 1). These insoluble domain proteins were therefore solubilised and purified in denaturing conditions using buffers containing 8 M urea.

For the purification of insoluble domain proteins from insoluble bacterial pellets, they were resuspended in Urea Loading Buffer (50 mM Tris at pH 8.0, 500 mM NaCl, 0.5% v/v Triton X-100, 8M urea) and left shaking for 1 h at room temperature. Supernatants were collected after 15 min centrifugation at 17,000 *g* at room temperature and then loaded onto Urea Loading Buffer equilibrated cobalt beads for 1 h incubation at room temperature. After 4 washes with Urea Wash Buffer (50 mM Tris at pH 8.0, 500 mM NaCl, 5 mM Imidazole, 0.5% v/v Triton X-100, 8M Urea), bound proteins were eluted with Urea Elution Buffer (50 mM Tris at pH 8, 500 mM NaCl, 200 mM Imidazole, 0.5% v/v Triton X-100, 8M Urea). Protein concentrations were quantified using a Bradford assay.

Supplementary Method 2 | Generation and culture of HEK293T FlpIn isogenic stable inducible cell lines.

Flp-In™ T-REx™ HEK293 parental cells (Invitrogen) were seeded at $1x10^6$ cells per 10 cm dish in 15 ml media (DMEM High Glucose, 10% Tet-Free FBS (Sigma), 1% PenStrep) in the presence of blasticidin S (Blast, 15 μg/ml, Calbiochem). Twenty-four hours post plating, cells were co-transfected with FLP recombinase plasmid (pGKFLPobpA, Addgene) and gene of interest (GOI) (pcDNA5/FRT/3xFLAG-GOI) in a ratio 6:4. Forty-eight hours post transfection, each confluent cell dish was split into 3x 10 cm dishes and cultured in 10 ml media mix (1:1 fresh:conditioned) in the absence of selection antibiotics. On the following day, hygromycin B (Hyg, 100 μg/ml, Invitrogen) was introduced into culture medium with Blast, and cells were cultured in Hyg/Blast selection medium for 7 days. The parental cell line carries dual resistance to blasticidin (near tetracycline repressor site) and zeocin (Zeo, 100 μg/ml, Invitrogen) at FRT site. During recombination, the zeocin resistance gene is replaced by the hygromycin resistance gene which is acquired from the integration of pcDNA5/FRT-GOI plasmids. Cells that have failed to incorporate GOI at FRT site die during the culture in the selection medium containing hygromycin. At day seven, medium was fully replaced with "fresh" media mix supplemented with Hyg/Blast and cells were cultured further for a week. During this period single colonies of cells carrying the GOI start to appear. Individual surviving colonies comprising of over 100 cells were then transferred to a single well on a 24-well plate and allowed to divide to confluency. At confluency, three-fifths of each well was transferred to a 6-well plate and cultured in media supplemented with Hyg/Blast. One-fifth of the cells were left in the same well and further cultured in Hyg/Blast selection medium while the remaining one-fifth was transferred to a neighbouring well and cultured in Zeo/Blast selection medium. Successful GOI incorporation was established when colonies survived in the media supplemented with Hyg/Blast but died in the presence of Zeo. Successful clones were further expanded to 10 cm dishes before being frozen down and stored in liquid nitrogen. Individual stable inducible cell lines were selected for downstream experiments after functional characterisation of the cells. The selection criteria were their ability to express GOI after doxycycline (1 μg/ml) induction for 72 h, normal doubling growth rate between 24-36 h, and healthy HEK293-like morphology.

For all experiments, cells were seeded in T175 flasks ($5x10^6$ cells), 10 cm dishes ($2x10^6$ cells), 6-well plates ($2x10^5$ cells) or 24-well plates (25,000 cells) in media plus Hyg/Blast. Cells were induced with 1 μg/ml doxycycline (Dox) 24 h post plating. For 6-day inductions, cells

were split 72 h after induction to maintain optimum confluency into fresh media plus Hyg /Blast and Dox. For galactose media experiments, cells were changed into galactose media 24 hours prior to assaying using Dulbecco's Modified Eagles Medium containing no glucose (Gibco™) supplemented with 10% TET-free FBS, 5 U/ml Penstrep, 5 mM galactose and Dox.

Supplementary Method 3 | Chromatin immunoprecipitation following sequencing (ChIP).

HEK FlpIn cell lines (Sham, PGC-1α WT-res and PGC-1α ΔRS-res) were cultured in 10 cm dishes and induced with Doxycycline for 6 days. Cells were collected in biological triplicates from three consecutive passages. ChIP-seq was performed based on the published STAR protocol[1] with minor modifications. Briefly, cells were washed once with DPBS prior to crosslinking with 2 mM Di(N-succinimidyl)-glutarate (DSG, Sigma-Aldrich, 80424) rocking at room temperature for 45 min. This was followed by three washes with DPBS and second crosslinking with 1% methanol-free formaldehyde (ThermoFisher, 28908) rocking at room temperature for 10 min. The reaction was quenched with 1:10 cold 1.25 M glycine for 5 min at room temperature. The crosslinking solution was removed and cells were scraped off in cold DPBS supplemented with 10% BSA. Pellet was collected by centrifugation at 2,000 *g* for 10 min at 4°C followed by two additional washes in cold DPBS with 5% BSA. After last wash, cell pellet was resuspended in 900 µl cold DPBS with 5% BSA, centrifuged one final time and snap-frozen in liquid nitrogen and kept at -80°C until being further processed. For ChIP, 100 µl Dynabeads Protein G (10004D, ThermoFisher) were incubated with 10 µg monoclonal anti-FLAG M2 mouse antibody (F1804, Sigma-Aldrich) in chromatin dilution buffer (25 mM Tris pH 7.5, 5 mM EDTA, 1% Triton X-100, 0.1% SDS) for 3 hours at room temperature. Meanwhile, cell pellets were resuspended in 300 µl SDS lysis buffer (50 mM Tris pH 7.5, 10 mM EDTA, 1% SDS) supplemented with protease inhibitors. Samples were transferred to Diagenode TGX tubes and sonicated using pre-chilled Diagenode Bioruptor Plus for 60 cycles (30 sec on, 30 sec off) on a high setting followed by dilution with 1 ml chromatin dilution buffer and centrifugation at 13,600 *g* for 30 min at 4°C. From supernatant, 5% was kept as input, while the rest was incubated with beads and antibody rotating overnight at 4°C. Dynabeads were then washed once with wash buffer A (50 mM HEPES pH 7.9, 140 mM NaCl, 1 mM EDTA, 1% Triton X-100, 0.1% sodium deoxycholate, 0.1% SDS), once with wash buffer B (50 mM HEPES pH 7.9, 500 mM NaCl, 1 mM EDTA, 1% Triton X-100, 0.1% sodium deoxycholate, 0.1% SDS), once with wash buffer C (20 mM Tris pH 8.0, 1 mM EDTA, 250 mM LiCl, 0.5% NP-40, 0.5% sodium deoxycholate), and twice with wash buffer TE (10 mM Tris pH 8.0, 1 mM EDTA). Immunoprecipitated protein:DNA were eluted twice with 100 µl elution buffer (10 mM Tris pH 7.5, 1 mM EDTA, 1% SDS) at 65°C for 5 min followed by 15 min at room temperature. Input samples were diluted to 200 µl with elution buffer. The

concentration of NaCl of all input and eluted samples was increased to 160 mM and 20 μg/ml RNase A (ThermoFisher) added followed by incubation at 65°C overnight. On the following day, EDTA was increased to 5 mM and proteins digested with 200 μg/ml Proteinase K (ThermoFisher) at 45°C for 2 hours. Genomic DNA was purified using ChIP DNA Clean and Concentrator kit (D5205, Zymo Research) following manufacturer's instructions.

NEB DNA Ultra II libraries were prepared manually following the manufacturer's protocol (NEBNext® Ultra™ II DNA Library Prep Kit for Illumina® Instruction Manual Version 5.0 6/18). Briefly, samples were normalized to 0.5 ng total DNA material per library in 25 μl of 10 mM Tris-HCl, pH 8.0.

Fragment end repair was carried out by adding 3.5 μl of End Prep Reaction Buffer and 1.5 μl of End Prep Enzyme Mix, then incubating for 30 minutes at 20°C followed by 30 minutes at 65°C. Adaptors were ligated to the repaired fragments by adding 15 μl Ligation Master Mix, 0.5 μl Ligation Enhancer and 1.25 μl Adaptor for Illumina (diluted to 0.6 μM), then incubating at 20°C for 15 minutes. To process adaptor ends, 1.5 μl of USER Enzyme was added to each sample and the reaction was incubated at 37°C for 15 minutes. Adaptor-ligated samples were cleaned up and size selected with SPRISelect beads (B23318; Beckman Coulter) for a final insert size of ~320 bp (ratio: 0.4x followed by 0.2x). For the amplification of the library, 12.5 μl of Q5 Master Mix was added, plus 5μl of each index (NEBNext Multiplex Oligos for Illumina (NEB #E6440S). The number of PCR cycles applied for library construction were 10 according to the manufacturer recommendation for 0.5 ng input DNA. The quality of the purified libraries was assessed using an Agilent D1000 ScreenTape Kit on an Agilent 4200 TapeStation. ChIP and Input samples were sequenced on the Illumina NovaSeq 6000 platform (Advanced Sequencing Facility, The Francis Crick Institute), and 100 bp single-end reads were generated.

Supplementary Method 4 | ChIP-seq analysis.

For sequence analysis, the nf-core/rnaseq, written in the Nextflow domain-specific language (ver. 22.04.0), was used to perform the primary analysis of the samples in conjunction with Singularity (ver. 3.6.4). The command used was “nextflow run nf-core/chipseq --narrow_peak --input design.csv -profile crick --genome GRCh38 --macs_fdr 0.05”. To summarize, the pipeline performs adapter trimming using TrimGalore!, read alignment using BWA, filtering using SAMtools, BEDTools, BamTools, pysam, and picard-tools, normalised coverage track generation using BEDTools and bedGraphToBig-Wig, peak calling using MACS, annotation relative to gene features using HOMER, consensus peak set creation using BEDTools, differential binding analysis using featureCounts and DESeq2, and extensive QC and version reporting using MultiQC, FastQC, prseq, deepTools, and phantompeakqualtools. All data were processed relative to the human GRCh38/hg38 genome and annotation. DNA binding was regarded as significant when PGC-1α or PGC-1α-ΔRS versus Sham was log2FC >0.5 and p-value <0.05. Promoters were standardly defined as the region encompassing 5,000 bp upstream and downstream of the transcription start site (TSS). The code is provided in Supplementary Figure 8.

Supplementary Method 5 | Next generation RNA sequencing.

HEK FlpIn cell lines (PGC-1α WT-res and PGC-1α ΔRS-res) were cultured in 6-well plates and induced with doxycycline for 6 days. Next generation RNA sequencing was carried out in biological triplicate experiments from three consecutive passages of cultured cells. For total (whole-cell) RNA, a single well was used, while 6 wells were pooled together for cytoplasmic RNA. Hypotonic lysis was used to isolate cytoplasmic fractions as outlined in the Methods section of the main text. Total (whole-cell) and fractionated extracts were added to PureZOL™ and RNA was extracted using Direct-zol RNA Miniprep Plus kit (Zymo Research). Extracted RNA was quality-checked on an Agilent 4200 TapeStation to ensure RNA integrity number (RIN) above 9.5. Sequencing libraries were prepared using the KAPA RNA HyperPrep Kit with RiboErase (HMR) (Roche, kit code KK8561) according to manufacturer's instructions. Briefly, samples were normalised to 600 ng of RNA in a final volume of 10 µl. Samples were specifically depleted for both cytoplasmic (5S, 5.8S, 18S, and 28S), and mitochondrial (12S and 16S) rRNA species, and eluted in 20 µl of Fragment, Prime and Elute Buffer. The fragmentation reaction was run for 6 min at 94°C for a library insert size of 200-300 bp. Fragmented RNA underwent first and second strand cDNA synthesis according to manufacturer's instructions. The KAPA Unique Dual-Indexed Adapters Kit (15 µM) (Roche KK8727) stock was diluted to 7 µM as recommended by the manufacturer for a total RNA input of 600 ng. The adaptor ligation was carried out by adding 5 µl of diluted adaptor and 45 µl of ligation mix (50 µl of ligation buffer + 10 µl of DNA ligase) to 60 µl of cDNA. The ligation reaction was run for 15 mins at 20°C. To remove short fragments such as adapter dimers, two SPRISelect bead clean-ups were done (0.63x SPRI and 0.7x SPRI). To amplify the library, 25 µl of Kapa HiFi HotStart PCR master mix plus 5 µl of Library Amplification Primer mix was added to 20 µl cDNA and 7 PCR cycles were performed as recommended by the manufacturer for a total RNA input of 600 ng. Amplified libraries were purified via a SPRISelect 1x bead clean-up. The quality and fragment size distributions of the purified libraries was assessed by a 4200 TapeStation Instrument (Agilent Technologies). Libraries were pooled and sequenced on the Illumina NovaSeq6000 in a 100 bp paired-end configuration (Advanced Sequencing Facility, The Francis Crick Institute).

Supplementary Method 6 | RNA-seq analysis.

For sequence analysis, the nf-core/rnaseq, written in the Nextflow domain-specific language (ver. 22.04.0), was used to perform the primary analysis of the samples in conjunction with Singularity (ver. 3.6.4). The command used was "nextflow run nf-core/rnaseq --input design.csv -profile crick --outdir ${PWD}/mouse --aligner star_rsem --fasta $GENOME_DIR/genome/Homo_sapiens.GRCh38.dna_sm.toplevel.fa --gtf Homo_sapiens.GRCh38.107.gtf.gz". Briefly, adapter and quality trimming was performed using Trim Galore!. The RSEM package in conjunction with the STAR alignment algorithm was used for the mapping, alignment and subsequent transcript-level counting of the sequenced reads with respect to the human GRCh38/hg38 genome and annotation.

An average of 151.7 millions 100 bp paired-end sequencing reads were aligned to the human genome (Supplementary Data 2). Approximately 10% of the genome is stably transcribed in human cells[2,3]. The size of the human genome is $3x10^9$ bp. Therefore according to the Lander/Waterman equation (Coverage = read length x number of reads / size of dataset): Transcriptome coverage = (2 x 100 bp x $151.7x10^6$ reads) / ($3x10^9$ bp x 10/100) = 101-fold. Principal component analysis (PCA) showed that replicates for the three whole-cell transcriptomes were well correlated, while it supported the removal of one replicate for each of the WT-res and ΔRS-res cytoplasmic transcriptome. Differential analysis was performed using DESeq2[4]. A p-value of <0.05 and a fold change of 0.5< log2 FC <-0.5 were used as the significance threshold for the identification of differentially expressed genes between the doxycycline-induced ΔRS-res and WT-res cell lines. Nuclear RNA export targets were identified as transcripts which are both (i) not differentially expressed in whole-cell transcriptomes (transcripts with 0.5< log2 FC <-0.5 and p-value < 0.05 were removed) and (ii) down-regulated in the cytoplasmic transcriptomes (log2 FC <-0.5 with p-value <0.05). Alternative splicing analysis was performed using Whippet[5]. The code is provided in Supplementary Figure 8.

Supplementary Method 7 | Tandem Mass tag (TMT) spectrometry and data processing.

HEK FlpIn cell (Sham, WT-res and ΔRS-res) were cultured in 6-well plates and induced with doxycycline for 6 days. TMT mass spectrometry was performed in biological triplicate experiments from three consecutive passages of cultured cells, washed with PBS containing protease inhibitors, gently scraped and flash frozen in liquid nitrogen followed by storage at -80°C. Cell pellets were lysed in Urea/SDS lysis buffer (8 M Urea, 1% SDS, 50 mM HEPES pH 8.2, 10 mM Glycerol-2-phosphate, 50 mM NaF, 5 mM Sodium pyrophosphate, 1 mM EDTA, 1 mM Sodium vanadate, 1 mM DTT, 500 nM Okadaic acid, protease inhibitors, Phosphatase inhibitor cocktail III). After protein concentration was measured using Pierce BCA assay, 100 μg of each sample was used for multiplexed quantitative proteomics giving a total of 1 mg protein. Each sample was reduced with 10 mM TCEP for 1 h at 55°C and alkylated with 20 mM IAA for 30 min in the dark at room temperature. In order to remove SDS, each reaction was precipitated with 6 volumes of cold (-20°C) acetone and left overnight at -20°C. Samples were equilibrated to room temperature to avoid urea crystals and centrifuged at 15,000 *g* for 10 min at room temperature. Supernatant was carefully removed and pellets were air-dried. Pellets were resuspended in 100 mM HEPES pH 8.5 by vortexing and incubation in a sonic bath for 15 min. Each resuspended sample was digested with 4 μg LysC (Lysyl endopeptidase, 125-05061, FUJIFILM Wako Chemicals) and 10 μg Trypsin (MS grade, 90058, ThermoFisher Scientific) at 37°C shaking overnight. Each sample was then tandem mass tag (TMT) labelled for an hour at room temperature using a TMT10plex Isobaric Label Reagent Set (0.8 mg per tag, 90110, ThermoFisher Scientific; LOT UK282322) and following manufacturer's instructions. A small aliquot of each sample was collected for a labelling efficiency and mixing accuracy quality checks (QCs) by liquid chromatography tandem mass spec (LC-MS/MS) using Orbitrap Fusion Lumos mass spectrometer and a 60 min HCD MS2 fragmentation method. The rest of the sample was stored at -80°C until QC results. Labelling efficiency of higher than 99% was obtained for each reaction and a mixing accuracy with lower than 1.5x difference between samples with lowest and highest summed intensity. Samples were defrosted, quenched with hydroxylamine for 15 min at room temperature and pooled together. Combined mixture was partially vacuum-dried and acidified to pH 2.0 followed by sample clean-up using C_{18} Sep Pak Vac 1cc, 50 ml bed volume (Waters) with gravity flow. Final mixing check was performed by LC-MS/MS with a 240 min HCD MS2 fragmentation method. From the pooled labelled mix, 100 μg total (starting) protein was aliquoted and vacuum-dried

to completion. Dried peptide mix was resuspended in 2% TFA and fractionated using Pierce High pH Reversed-Phase Fractionmation kit (84868, ThermoFisher Scientific). Each fraction was vacuum-dried and resuspended in 25 µl 0.1% TFA. Peptides were separated on a 50 cm, 75 µm I.D. Pepmap column over a 2 h gradient and eluted directly into the mass spectrometer (Orbitrap Fusion Lumos) with 3 injections of 7 µl for each fragmentation method – HCD MS2, and two methods for MS3[6,7]. Xcalibur software was used to control the data acquisition. The instrument was run in data dependent acquisition mode with the most abundant peptides selected for MS/MS by HCD fragmentation.

Raw data were processed using MaxQuant v1.6.2.10 and Uniprot human reference proteome from September 2020. Processed data were then analysed using an R-coding script tailored to isobaric labelling mass spectrometry. The script was generated as a hybrid using the backbone and differential gene expression analysis of ProteoViz package[8] as a general script workflow and borrowing the normalization script from Proteus package[8]. Briefly, the "proteinGroups.txt" table was read into matrices and filtered for "reverse" hits, "potential contaminant" and proteins "only identified by site". Data were then normalized using CONSTANd[9], log2-transformed and differentially analysed using Linear Models for Microarray Data (limma). Significant hits were called proteins with adjusted p-value < 0.05. Volcano plots were generated using ggrepel (a ggplot2 extension) as part of the tidyverse. Venn diagram was generated using the VennDiagram package. The code is provided in Supplementary Figure 8.

Supplementary Method 8 | Codes used for the bioinformatics analysis.

RNA-seq:

Run nf-core/rnaseq pipeline

The nf-core rnaseq pipeline was run like:

```
#!/usr/bin/bash


module purge

ml Nextflow/22.04.0

ml Singularity/3.6.4

ml Graphviz


export NXF_SINGULARITY_CACHEDIR=/path/to/singularity/cache


cd /path/to/project/working/directory


GENOME_DIR=/camp/svc/reference/Genomics/babs/homo_sapiens/ensembl/GRCh38/re
lease-95/


nextflow run nf-core/rnaseq --input design.csv -profile crick --outdir ${PW
D}/mouse --aligner star_rsem --fasta $GENOME_DIR/genome/Homo_sapiens.GRCh38
.dna_sm.toplevel.fa --gtf Homo_sapiens.GRCh38.107.gtf.gz
```

Set up R for this analysis:

```
library(DESeq2)

library(apeglm)

library(ashr)

library(EnhancedVolcano)

library(IHW)

library(mixsqp)

library(stringr)

library(org.Hs.eg.db)

library(fgsea)

library(msigdbr)
```

```
library(tidyr)
library(tibble)
library(openxlsx)
library(ggVennDiagram)
library(readr)
library(pheatmap)
library(data.table)
```

Set the working directory

```
setwd('/Volumes/babs/working/goldstr2/projects/ultanirs/simeon.mihaylov/RN22132-sm256/analysis')
```

Reading in the design file

This file just specifies which samples belong to which group. I have saved this design file in the project folder. Using this file, I will re-order the columns of ddm to make sure the rownames of coldata match the column names of ddm

```
coldata <- read.csv('samplesheet.csv')

# to compare between groups, we need to make a new column that joins genotype and sub-cellular compartment

coldata$group <- paste(coldata$genotype, coldata$subcellular_compartment, sep="_")

knitr::kable(coldata)
```

ID	sample	genotype	subcellular_compartment	cell_pellet_id	group
MIH4868A19	PGC1a_ WT-res _total_Rep1_NEW	WT-res	whole_cell	PGC1a_ WT-res _Rep1_NEW	WT-res _whole_cell
MIH4868A21	PGC1a_ WT-res _total_Rep2_NEW	WT-res	whole_cell	PGC1a_ WT-res _Rep2_NEW	WT-res _whole_cell
MIH4868A23	PGC1a_ WT-res _total_Rep3_NEW	WT-res	whole_cell	PGC1a_ WT-res _Rep3_NEW	WT-res _whole_cell
MIH4868A25	PGC1a_ WT-res _cyto_Rep1_NEW	WT-res	cytoplasm	PGC1a_ WT-res _Rep1_NEW	WT-res _cytoplasm
MIH4868A27	PGC1a_ WT-res _cyto_Rep2_NEW	WT-res	cytoplasm	PGC1a_ WT-res _Rep2_NEW	WT-res _cytoplasm

MIH4868A29	PGC1a_ WT-res _cyto_Rep3_NEW	WT-res	cytoplasm	PGC1a_ WT-res _Rep3_NEW	WT-res _cytoplasm
MIH4868A20	PGC1a_ΔRS-res _total_Rep1_NEW	ΔRS-res	whole_cell	PGC1a_ΔRS-res _Rep1_NEW	PGC1a_ΔRS-res _whole_cell
MIH4868A22	PGC1a_ΔRS-res _total_Rep2_NEW	ΔRS-res	whole_cell	PGC1a_ΔRS-res _Rep2_NEW	PGC1a_ΔRS-res _whole_cell
MIH4868A24	PGC1a_ΔRS-res _total_Rep3_NEW	ΔRS-res	whole_cell	PGC1a_ΔRS-res _Rep3_NEW	PGC1a_ΔRS-res _whole_cell
MIH4868A26	PGC1a_ΔRS-res _cyto_Rep1_NEW	ΔRS-res	cytoplasm	PGC1a_ΔRS-res _Rep1_NEW	PGC1a_ΔRS-res _cytoplasm
MIH4868A28	PGC1a_ΔRS-res _cyto_Rep2_NEW	ΔRS-res	cytoplasm	PGC1a_ΔRS-res _Rep2_NEW	PGC1a_ΔRS-res _cytoplasm
MIH4868A30	PGC1a_ΔRS-res _cyto_Rep3_NEW	ΔRS-res	cytoplasm	PGC1a_ΔRS-res _Rep3_NEW	PGC1a_ΔRS-res _cytoplasm

Reading in the data as a matrix

```
dd <- read.csv('../results/star_rsem/rsem.merged.transcript_counts.tsv',sep
="\t")

rownames(dd) <- dd[,1]

ddm <- as.matrix(dd[,-c(1,2)])

ddm  <- round(ddm)
```

Translate the ENSEMBL IDs to gene symbols.

Read in the annotation GTF to use to translate the ENSEMBL IDs to gene symbols.

```
gtf <- rtracklayer::import('/camp/stp/babs/working/goldstr2/gtf/GRCh38/107/
Homo_sapiens.GRCh38.107.gtf.gz')

names(gtf) <- gtf$gene_id
```

ChIP-seq:

Run nf-core/chipseq pipeline

The nf-core chipseq pipeline was run like:

```
#!/usr/bin/bash

module purge
ml Nextflow/22.04.0
ml Singularity/3.6.4
ml Graphviz

export NXF_SINGULARITY_CACHEDIR=/path/to/singularity/cache

cd /path/to/project/working/directory

nextflow run nf-core/chipseq --narrow_peak --input design.csv -profile cric
k --genome GRCh38  --macs_fdr 0.05
```

Set the working directory

```
setwd('/Volumes/babs/working/goldstr2/projects/ultanirs/simeon.mihaylov/PM2
2133-sm258/analysis/version_3')
```

Read the samplesheet for DiffBind

To do the analysis, we'll make use of an R package called DiffBind. we need to pass DiffBind a samplesheet that tells it what the samples / inputs are and where the files exist.

```
dd <- read.csv('../diffbind_samplesheet.csv')

knitr::kable(dd)
```

sampleID	Tissue	Factor	Treatment	Replicate	bamReads	ControlID	bamControl	Peaks	PeakCaller
Sham_ChI	Sham	NA	NA	1	/camp/stp/babs/working/goldstr2/projects/ultanirs/simeon.mihayl	Sham_INPU	/camp/stp/babs/working/goldstr2/projects/ultanirs/simeon.mihayl	/camp/stp/babs/working/goldstr2/projects/ultanirs/simeon.mihaylov/PM22	macs

P_R1					ov/PM22133-sm258/results/bwa/mergedLibrary/Sham_ChIP_R1.mLb.clN.sorted.bam	T_R1	ov/PM22133-sm258/results/bwa/mergedLibrary/Sham_INPUT_R1.mLb.clN.sorted.bam	133-sm258/results/bwa/mergedLibrary/macs/narrowPeak/Sham_ChIP_R1_peaks.narrowPeak	
PGC1a_WT-res_ChIP_R1	PGC1a_WT-RES	NA	NA	1	/camp/stp/babs/working/goldstr2/projects/ultanirs/simeon.mihaylov/PM22133-sm258/results/bwa/mergedLibrary/PGC1a_WT-RES_ChIP_R1.mLb.clN.sorted.bam	PGC1a_WT-RES_INPUT_R1	/camp/stp/babs/working/goldstr2/projects/ultanirs/simeon.mihaylov/PM22133-sm258/results/bwa/mergedLibrary/PGC1a_WT-RES_INPUT_R1.mLb.clN.sorted.bam	/camp/stp/babs/working/goldstr2/projects/ultanirs/simeon.mihaylov/PM22133-sm258/results/bwa/mergedLibrary/macs/narrowPeak/PGC1a_WT-RES_ChIP_R1_peaks.narrowPeak	macs
PGC1a_ΔRS-res_ChIP_R1	PGC1a_ΔRS-res	NA	NA	1	/camp/stp/babs/working/goldstr2/projects/ultanirs/simeon.mihaylov/PM22133-sm258/results/bwa/mergedLibrary/PGC1a_ΔRS-res_ChIP_R1.mLb.clN.sorted.bam	PGC1a_ΔRS-res_INPUT_R1	/camp/stp/babs/working/goldstr2/projects/ultanirs/simeon.mihaylov/PM22133-sm258/results/bwa/mergedLibrary/PGC1a_ΔRS-res_INPUT_R1.mLb.clN.sorted.bam	/camp/stp/babs/working/goldstr2/projects/ultanirs/simeon.mihaylov/PM22133-sm258/results/bwa/mergedLibrary/macs/narrowPeak/PGC1a_ΔRS-res_ChIP_R1_peaks.narrowPeak	macs
Sham_ChIP_R2	Sham	NA	NA	2	/camp/stp/babs/working/goldstr2/projects/ultanirs/simeon.mihaylov/PM22133-sm258/results/bwa/mergedLibrary/Sham_ChIP_R2.mLb.clN.sorted.bam	Sham_INPUT_R2	/camp/stp/babs/working/goldstr2/projects/ultanirs/simeon.mihaylov/PM22133-sm258/results/bwa/mergedLibrary/Sham_INPUT_R2.mLb.clN.sorted.bam	/camp/stp/babs/working/goldstr2/projects/ultanirs/simeon.mihaylov/PM22133-sm258/results/bwa/mergedLibrary/macs/narrowPeak/Sham_ChIP_R2_peaks.narrowPeak	macs
PGC1a_WT-RES_ChIP_R2	PGC1a_WT-RES	NA	NA	2	/camp/stp/babs/working/goldstr2/projects/ultanirs/simeon.mihaylov/PM22133-sm258/results/bwa/mergedLibrary/PGC1a_WT-RES_ChIP_R2.mLb.clN.sorted.bam	PGC1a_WT-RES_INPUT_R2	/camp/stp/babs/working/goldstr2/projects/ultanirs/simeon.mihaylov/PM22133-sm258/results/bwa/mergedLibrary/PGC1a_WT-RES_INPUT_R2.mLb.clN.sorted.bam	/camp/stp/babs/working/goldstr2/projects/ultanirs/simeon.mihaylov/PM22133-sm258/results/bwa/mergedLibrary/macs/narrowPeak/PGC1a_WT-RES_ChIP_R2_peaks.narrowPeak	macs
PGC1a_ΔRS-res_ChIP_R2	PGC1a_ΔRS-res	NA	NA	2	/camp/stp/babs/working/goldstr2/projects/ultanirs/simeon.mihaylov/PM22133-sm258/results/bwa/mergedLibrary/PGC1a_□RS-res_ChIP_R2.mLb.clN.sorted.bam	PGC1a_ΔRS-res_INPUT_R2	/camp/stp/babs/working/goldstr2/projects/ultanirs/simeon.mihaylov/PM22133-sm258/results/bwa/mergedLibrary/PGC1a_ΔRS-res_INPUT_R2.mLb.clN.sorted.bam	/camp/stp/babs/working/goldstr2/projects/ultanirs/simeon.mihaylov/PM22133-sm258/results/bwa/mergedLibrary/macs/narrowPeak/PGC1a_ΔRS-res_ChIP_R2_peaks.narrowPeak	macs
Sham_ChIP_R3	Sham	NA	NA	3	/camp/stp/babs/working/goldstr2/projects/ultanirs/simeon.mihaylov/PM22133-sm258/results/bwa/mergedLibrary/Sham_C	Sham_INPUT_R3	/camp/stp/babs/working/goldstr2/projects/ultanirs/simeon.mihaylov/PM22133-sm258/results/bwa/mergedLibrary/Sham_I	/camp/stp/babs/working/goldstr2/projects/ultanirs/simeon.mihaylov/PM22133-sm258/results/bwa/mergedLibrary/macs/narrowP	macs

					hIP_R3.mLb.clN.sorted.bam		NPUT_R3.mLb.clN.sorted.bam	eak/Sham_ChIP_R3_peaks.narrowPeak	
PGC1a_WT-RES_ChIP_R3	PGC1a_WT-RES	NA	NA	3	/camp/stp/babs/working/goldstr2/projects/ultanirs/simeon.mihaylov/PM22133-sm258/results/bwa/mergedLibrary/PGC1a_WT-RES_ChIP_R3.mLb.clN.sorted.bam	PGC1a_WT-RES_INPUT_R3	/camp/stp/babs/working/goldstr2/projects/ultanirs/simeon.mihaylov/PM22133-sm258/results/bwa/mergedLibrary/PGC1a_WT-RES_INPUT_R3.mLb.clN.sorted.bam	/camp/stp/babs/working/goldstr2/projects/ultanirs/simeon.mihaylov/PM22133-sm258/results/bwa/mergedLibrary/macs/narrowPeak/PGC1a_WT-RES_ChIP_R3_peaks.narrowPeak	macs
PGC1a_ΔRS-res_ChIP_R3	PGC1a_ΔRS-res	NA	NA	3	/camp/stp/babs/working/goldstr2/projects/ultanirs/simeon.mihaylov/PM22133-sm258/results/bwa/mergedLibrary/PGC1a_□RS-res_ChIP_R3.mLb.clN.sorted.bam	PGC1a_ΔRS-res_INPUT_R3	/camp/stp/babs/working/goldstr2/projects/ultanirs/simeon.mihaylov/PM22133-sm258/results/bwa/mergedLibrary/PGC1a_ΔRS-res_INPUT_R3.mLb.clN.sorted.bam	/camp/stp/babs/working/goldstr2/projects/ultanirs/simeon.mihaylov/PM22133-sm258/results/bwa/mergedLibrary/macs/narrowPeak/PGC1a_ΔRS-res_ChIP_R3_peaks.narrowPeak	macs

```
samples <- dba(sampleSheet=dd)

samples <- dba.count(samples, bParallel = F)

samples <- dba.normalize(samples, background = TRUE, library = DBA_LIBSIZE_DEFAULT)

samples <- dba.contrast(samples,design = "~ Tissue", reorderMeta=list(Tissue="PGC1a_WT-RES"))

samples <- dba.analyze(samples, bParallel = F)
```

Proteomics:

1) Sample metadata:

Sample	Enrichment	Batch	Replicate	Pool	Sample_name	Model_group
Reporter_intensity_corrected_1	Lysate	0	1		WT-res GLU 1	WT-res
Reporter_intensity_corrected_2	Lysate	0	2		WT-res GLU 2	WT-res
Reporter_intensity_corrected_3	Lysate	0	3		WT-res GLU 3	WT-res
Reporter_intensity_corrected_4	Lysate	0	4		WT-res GAL 1	WT-res
Reporter_intensity_corrected_5	Lysate	0	5		WT-res GAL 2	WT-res
Reporter_intensity_corrected_6	Lysate	0	1		ΔRS-res GLU 1	ΔRS-res
Reporter_intensity_corrected_7	Lysate	0	2		ΔRS-res GLU 2	ΔRS-res
Reporter_intensity_corrected_8	Lysate	0	3		ΔRS-res GLU 3	ΔRS-res
Reporter_intensity_corrected_9	Lysate	0	4		ΔRS-res GAL 1	ΔRS-res
Reporter_intensity_corrected_10	Lysate	0	5		ΔRS-res GAL 2	ΔRS-res

2) Contrast matrix:

Contrast_name
ΔRS-res – WT-res

3) Make metadata

```
library(tidyverse)

if(!dir.exists("data")){dir.create("data")}


df <- read_tsv("txt/proteinGroups.txt", guess_max = 10000) %>%
{set_names(., gsub(" ", "_", names(.)))}


df %>%
  select(Majority_protein_IDs, Fasta_headers, Score, id, `Phospho_(STY)_site_IDs`) %>%
  mutate(Description = str_extract(Fasta_headers, "(?<= )[^\\|]+(?= OS\\=)"),
         Gene_name = str_extract(Fasta_headers, "(?<=GN\\=)[^\\|]+(?= PE\\=)"),
         Uniprot_ID = str_extract(Fasta_headers, "(?<=\\|)[^\\|]+(?=\\|)")) %>%
```

```
  write_tsv("data/Protein_metadata.tsv")

phos_df <- read_tsv("txt/Phospho (STY)Sites.txt", guess_max = 20000) %>%
{set_names(., gsub(" ", "_", names(.)))}

phos_df %>%
  select(Proteins:Score, Amino_acid, Sequence_window, `Phospho_(STY)_Probabilities`,
Charge, id:Evidence_IDs) %>%
  mutate(Description = str_extract(Fasta_headers, "(?<= )[^\\|]+(?= OS\\=)"),
        Gene_name = str_extract(Fasta_headers, "(?<=GN\\=)[^\\|]+(?= PE\\=)"),
        Uniprot_ID = str_extract(Fasta_headers, "(?<=\\|)[^\\|]+(?=\\|)"),
        Flanking = gsub("\\;.*$", "", Sequence_window) %>%
          str_sub(9,23) %>%
          paste0("-p")) %>%
  write_tsv("data/Phospho_metadata.tsv")
```

4) Total proteome analysis

```
library(tidyverse)
library(limma)


df <- read_tsv("txt/proteinGroups.txt", guess_max = 20000) %>%
  {set_names(., gsub(" ", "_", names(.)))}

sample_df <- read_tsv("Sample_metadata.tsv") %>%
  mutate(Sample_name = factor(Sample_name, levels = unique(Sample_name)),
        Model_group = factor(Model_group, levels = unique(Model_group))) %>%
  #For protein
  filter(grepl("Lysate", Enrichment))

contrast_df <- read_tsv("contrast_matrix.tsv")

df1 <- df %>%
  filter(is.na(Reverse),
        is.na(Potential_contaminant),
        is.na(Only_identified_by_site))

df1[93:102][df1[93:102] == 0] <- NA

# using RAS (constrained standardisation CONSTANd) to normalise data
# the mean in each column and row equals to 1/n, where n is the number of columns and
rows, respectively
# this bit of code was taken from Proteus package
RAS <- function(K, max.iter=50, eps=1e-5) {
  n <- ncol(K)
  m <- nrow(K)

  # ignore rows with only NAs
```

```
  good.rows <- which(rowSums(!is.na(K)) > 9)
  K <- K[good.rows, ]

  cnt <- 1
  repeat {
    row.mult <- 1 / (n * rowMeans(K, na.rm=TRUE))
    K <- K * row.mult
    err1 <- 0.5 * sum(abs(colMeans(K, na.rm=TRUE) - 1/n))
    col.mult <- 1 / (n * colMeans(K, na.rm=TRUE))
    K <- t(t(K) * col.mult)
    err2 <- 0.5 * sum(abs(rowMeans(K, na.rm=TRUE) - 1/n))
    cnt <- cnt + 1
    if(cnt > max.iter || (err1 < eps && err2 < eps)) break
  }

  # reconstruct full table
  KF <- matrix(NA, nrow=m, ncol=n)
  KF[good.rows, ] <- K

  return(KF)
}

normalizeTMT <- function(datf, max.iter=50, eps=1e-5) {{
  datf[93:102] <- RAS(datf[93:102])
}
  return(datf)
}

df1n <- normalizeTMT(df1)

df2 <- df1n %>%
  mutate_at(vars(matches("corrected.*")), log2)

df2a <- df2 %>%
  select(id, one_of(sample_df$Sample)) %>%
  gather(Sample, Intensity, one_of(sample_df$Sample))

df2b <- df2a %>%
  right_join(sample_df) %>%
  group_by(id, Batch) %>%
  group_by(Sample) %>%
  ungroup()

df2b %>%
  select(-Sample) %>%
  write_tsv("data/Normalized_proteingroup_intensities.tsv")



# Limma ----------------------------------------------------------------
```

```
# Make model matrix and contrasts

model_df <- sample_df %>%
  filter(!is.na(Sample_name))

design <- model.matrix(~ 0 + model_df$Model_group)
colnames(design) <- unique(model_df$Model_group)


cont_table <- makeContrasts(contrasts = as.list(contrast_df$Contrast_name), levels =
model_df$Model_group)
Comparisons <- dimnames(cont_table)$Contrasts

# Format for Limma

df3 <- df2b %>%
  filter(!is.na(Sample_name)) %>%
  select(id, Sample_name, Intensity) %>%
  spread(Sample_name, Intensity) %>%
  as.data.frame() %>%
  write_tsv("data/Protein_limma_input.tsv") %>%
  column_to_rownames("id")

fit <- lmFit(df3, design)

cont_fit <- contrasts.fit(fit, cont_table)

fit2 <- eBayes(cont_fit)


#Extract topTable for each contrast
f1 <- function(x1){
  a1_name <- colnames(fit2$coefficients)[[x1]]
  a1 <- topTable(fit2, x1, number = Inf) %>%
    rownames_to_column("id") %>%
    mutate(Comparison = a1_name) %>%
    mutate(id = as.integer(id)) %>%
    as_tibble() %>%
    left_join(df %>% select(id),
              by = "id")

  a1
}

df4 <- map_df(seq_along(Comparisons), f1) %>%
  filter(!is.na(adj.P.Val))

df4 %>%
  write_tsv("data/Protein_limma_output.tsv")
```

```
spread_protein_limma <- df4 %>%
  select(id, logFC, adj.P.Val, P.Value, Comparison) %>%
  gather(Type, Value, logFC, adj.P.Val, P.Value) %>%
  unite(Type, Comparison, Type, sep = " ") %>%
  spread(Type, Value) %>%
  mutate(id = as.integer(id))

protein_meta <- read_tsv("data/Protein_metadata.tsv")

protein_quantitative <- df3 %>%
  rownames_to_column("id") %>%
  mutate(id = as.integer(id))


#For filtering, only include proteins identified in at least 1 sample
samples <- as.character(na.omit(sample_df$Sample_name))

summarized_protein <- protein_meta %>%
  left_join(protein_quantitative) %>%
  left_join(spread_protein_limma) %>%
  filter_at(vars(one_of(samples)), any_vars(!is.na(.))) %>%
  write_tsv("data/Protein_summarized_data.tsv")
```

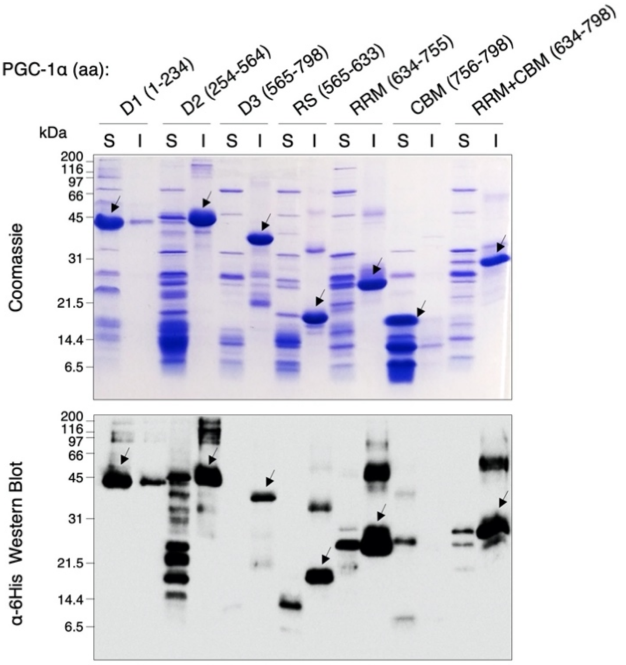

Supplementary Fig. 1 | Recombinant expression and purification screening of PGC-1α domains. GB1-6His-tagged protein domains were expressed in *E. coli* BL21 for 3 hours at 37˚C prior to small batch purification of soluble (S) and insoluble (I) proteins onto TALON/Cobalt-loaded beads. Eluted proteins were analysed by SDS-PAGE stained with Coomassie blue (top panel) and western blot using α-6His antibodies (bottom panel). Protein domains of PGC-1α are indicated with aa in brackets depicting the amino-acids number positions. 5 µg purified proteins were loaded in each lane. Arrows indicate full length protein domains. Similar results were obtained in at least three independent experimental settings.

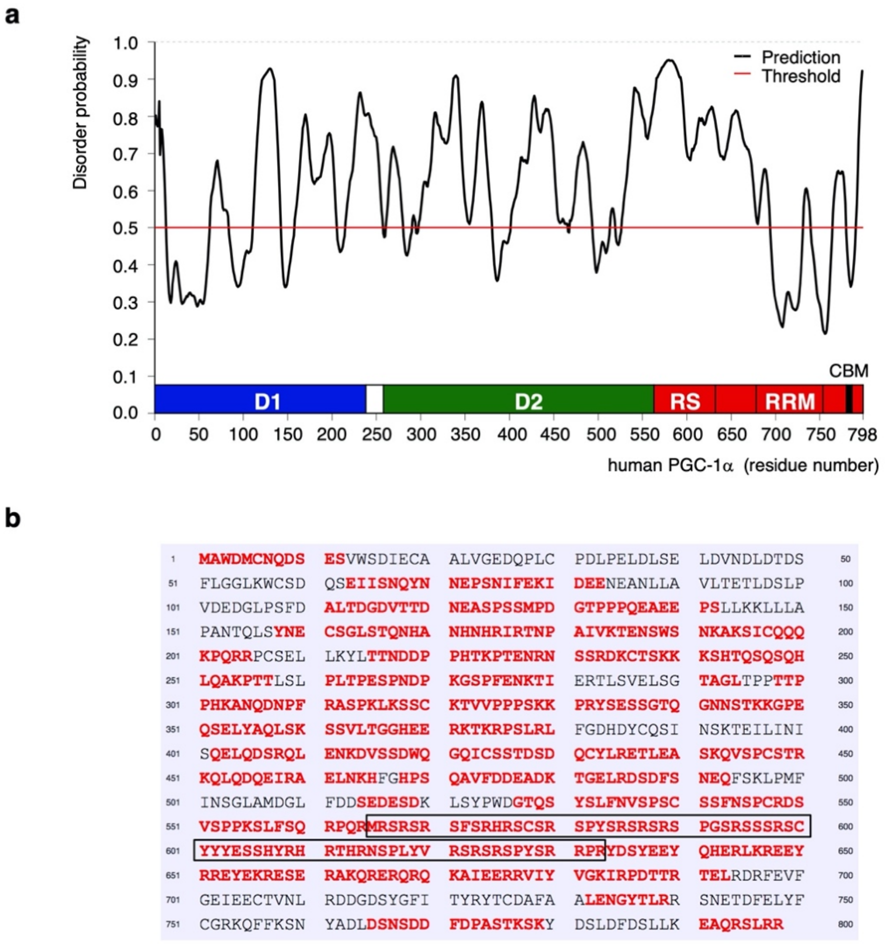

Supplementary Fig. 2 | The RS domain of PGC-1α is predicted to be unstructured. (a) Probability of disorder is plotted in function of the amino-acid sequence of human PGC-1α using the Protein DisOrder prediction System (PrDOS: https://prdos.hgc.jp/cgi-bin/top.cgi) using a prediction false positive rate of 5%. **(b)** Disordered residues are labelled in red. They indicate that a large proportion of the protein is unstructured in agreement with the finding that PGC-1α belongs to the family of intrinsically disordered proteins with a fast turnover mediated by the 20S proteasome[30]. The RS domain is highlighted with black boxes (amino acids 565-633).

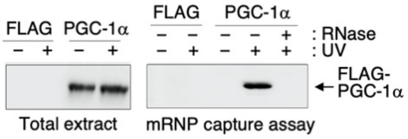

Supplementary Fig. 3 | PGC-1α interacts with polyadenylated RNA. HEK293T cells were transfected for 48 hours with either FLAG control, FLAG-tagged PGC-1α wildtype or FLAG-tagged PGC-1α ΔRS. Cells were subjected to UV-irradiation (+) or not (-) prior to RNase treatment of protein extracts (where indicated) and denaturing mRNP capture assays using oligo-dT beads. Eluted proteins were analysed by western blotting probed with α-FLAG antibody. This experiment with RNAse treatment was performed once, however the mRNP capture assays presented in Fig. 1d were performed in 3 independent experiments.

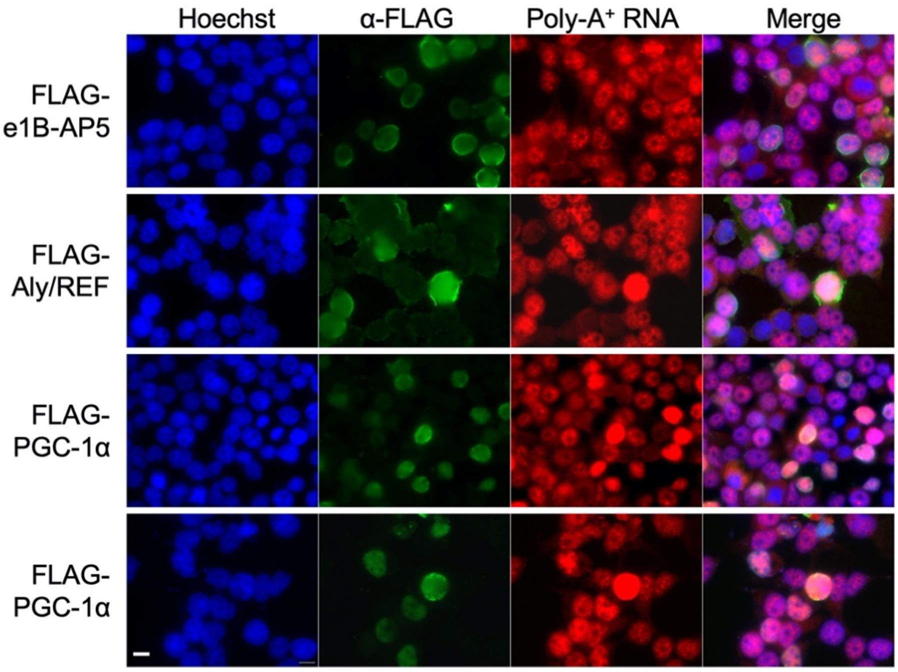

Supplementary Fig. 4 | Cellular distribution of bulk poly(A)+ RNAs upon overexpression of PGC-1α. HEK293T cells were transfected with E1B-AP5/hnRNPUL1 (negative control), Aly/REF (positive mRNA export adaptor control) and PGC-1α for 48 hours followed by a 2 h treatment with 5 µg/ml Actinomycin D. Cells were stained with anti-FLAG antibody (green) to visualise transfected cells and Cy3-oligo(dT) (red) that anneal to the poly(A) tails of RNA molecules. Cells with overexpression of FLAG-tagged Aly/REF and PGC-1α show a block in the bulk nuclear export of mRNAs with saturated accumulation of Poly-A+ RNA nuclear staining and absence of detectable cytoplasmic signal. Scale bar: 10 µm. Similar results were obtained in three independent experimental settings.

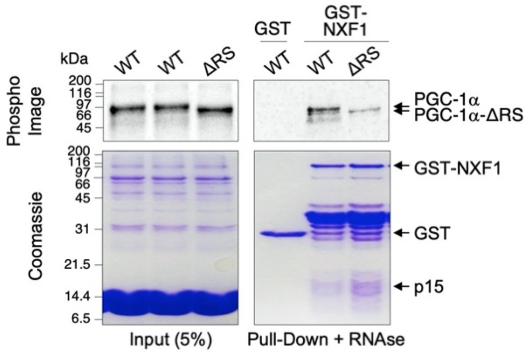

Supplementary Fig. 5 | Deletion of the RS domain of PGC-1α alters its interaction with NXF1. GST pull down assays. Bacterially-expressed GST or GST-NXF1:p15 bound to glutathione-coated beads were incubated with ^{35}S-PGC-1α WT and ΔRS radiolabelled in rabbit reticulocytes in the presence of RNase. Coomassie images show input and pull-down with immobilised GST and GST-NXF1:p15. PhoshoImages show ^{35}S-radiolabelled PGC-1α input (left) and pulldown (right) lanes. This experiment was performed once.

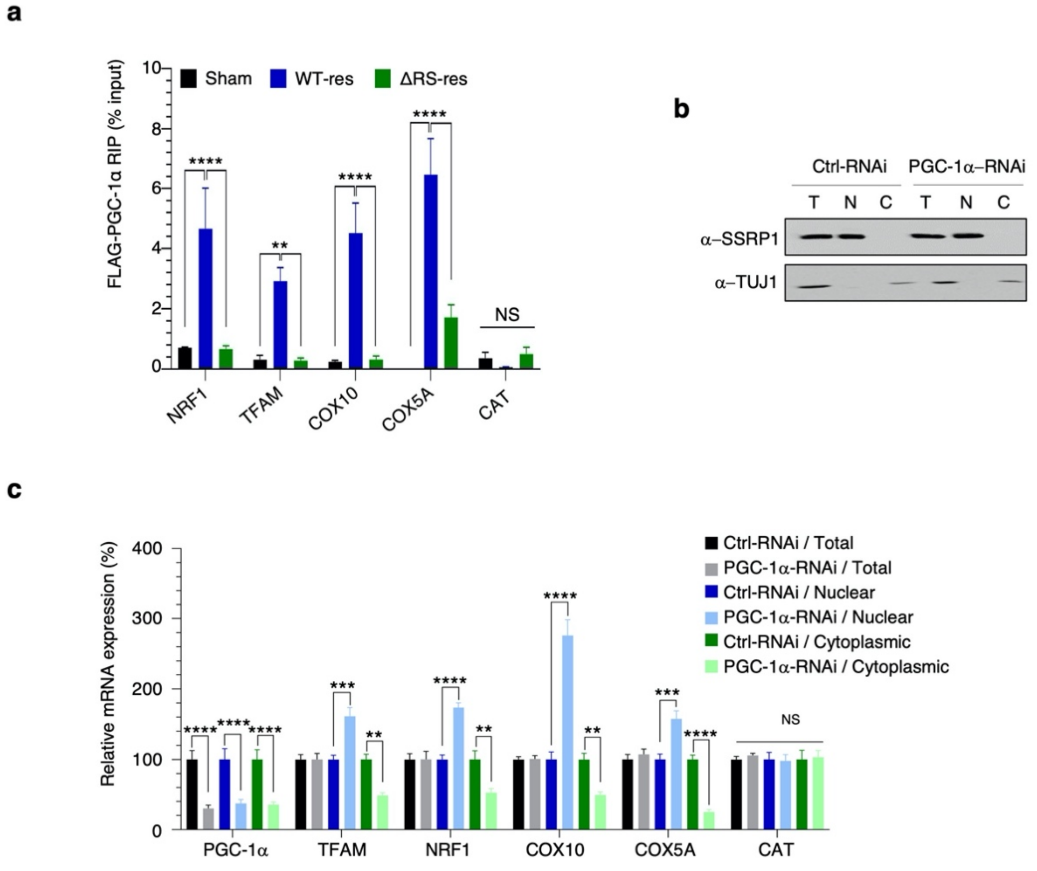

Supplementary Fig. 6 | PGC-1α plays a role in the mRNA nuclear export pathway. (**a**) Formaldehyde cross-linked RNA immunoprecipitation (RIP) assays from Sham, FLAG-tagged RNAi-resistant PGC-1α WT (WT-res) and FLAG-tagged PGC-1α-ΔRS (ΔRS-res) human stable cell lines induced for 6 days with doxycycline and subjected to anti-FLAG immunoprecipitation. Purified RNA was analysed by qRT-PCR and expressed as a percentage of the input. RIPs were performed in three biological replicate experiments (Mean ± SEM; two-way ANOVA with Turkey's correction for multiple comparisons; NS: not significant, **: p<0.01, ****: p<0.0001; N=3). (**b**) Western blots of Ctrl-RNAi and PGC-1α-RNAi cell lines induced for 6 days with doxycycline and cultured in galactose media for the last 24 h prior to cellular fractionation using hypotonic lysis to yield cytoplasmic fractions. The chromatin-remodeling SSRP1 factor is used to check for potential nuclear contamination in cytoplasmic fractions. Depletion of beta-Tubulin III (TUJ1) in nuclear fractions was used to check for quality of the nuclear fractions. (**c**) Total, nuclear and cytoplasmic levels of fractionated RNA were quantified in three biological replicate experiments by qRT-PCR following normalisation to U1 snRNA levels and to 100% in Ctrl-RNAi cell line (Mean ± SEM; two-way ANOVA with Tukey's correction for multiple comparisons, NS: not significant, **: p<0.01, ***: p<0.001, ****: p<0.0001; N=3). Source data with details of statistical tests are provided with exact p-values as a Source Data file.

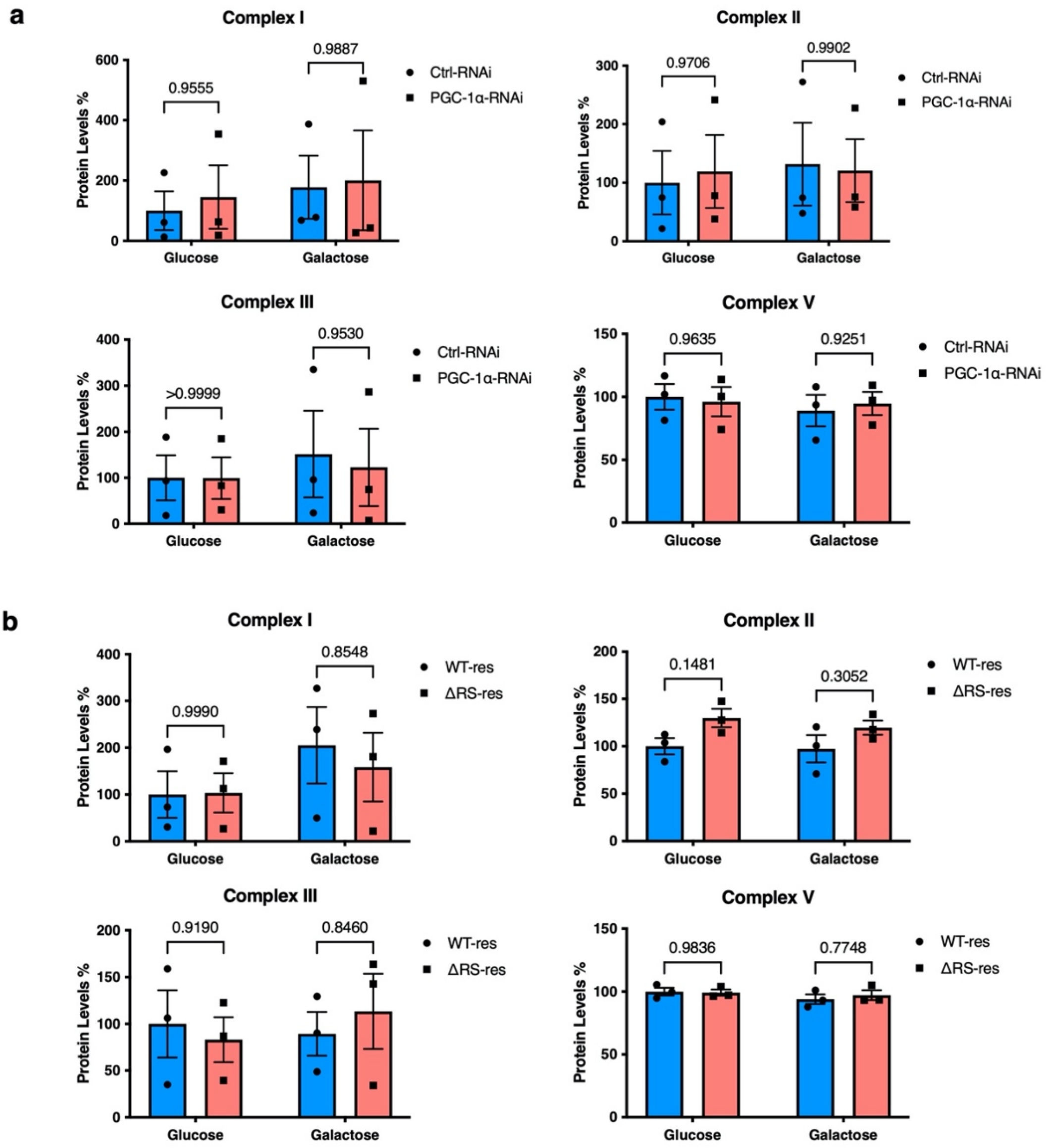

Supplementary Fig. 7 | Quantification of OXPHOS western blots in figure 6a-b. (a) OXPHOS western blots shown in figure 6a were performed in three independent experiments and changes in protein levels quantified and normalised against the total mitochondrial protein level (the sum of all 5 complexes) (Mean ± SEM; two-way ANOVA with Šidák's correction for multiple comparisons; All not significant, adjusted p-value is indicated above each comparison). **(b)** OXPHOS western blots shown in figure 6a were performed in three independent experiments and changes in protein levels quantified and normalised against the total mitochondrial protein level (the sum of all 5 complexes) (Mean ± SEM; two-way ANOVA with Šidák's correction for multiple comparisons; adjusted p-value is indicated above each comparison). Source data with details of statistical tests are provided as a Source Data file.

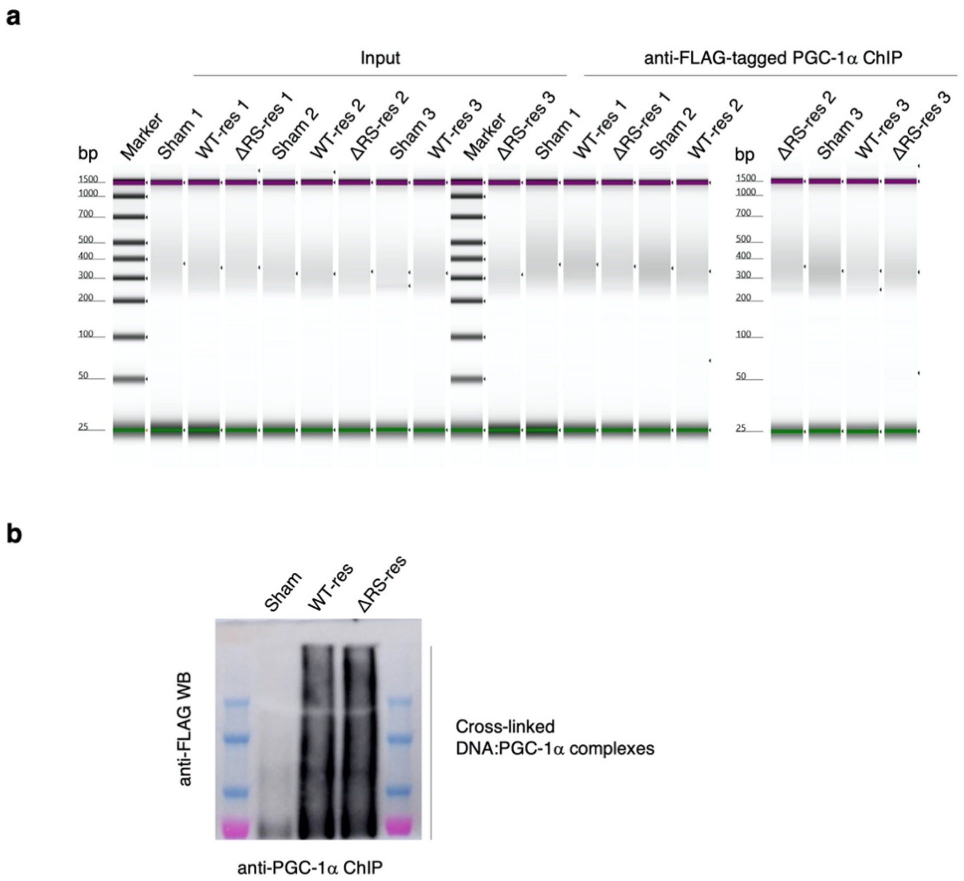

Supplementary Fig. 8 | Chromatin immunoprecipitation followed by sequencing (ChIP-seq). (**a**) 6-day doxycycline-induced Sham, WT-res and ΔRS-res cell lines were subjected to DRG/formaldehyde cross-linking prior to shearing of the chromatin by sonication using a *Diagenode Bioruptor Plus* (60 cycles 30 sec on/ 30 sec off on a high setting). Bioanalyser traces show that sizes of sheared chromatin fragments peak around 300/400 bp in both input and eluted ChIP samples. (**b**) Eluted anti-FLAG-tagged PGC-1α ChIP samples were analysed by western blotting (WB) probed with α-FLAG antibody. Three independent experiments were performed.

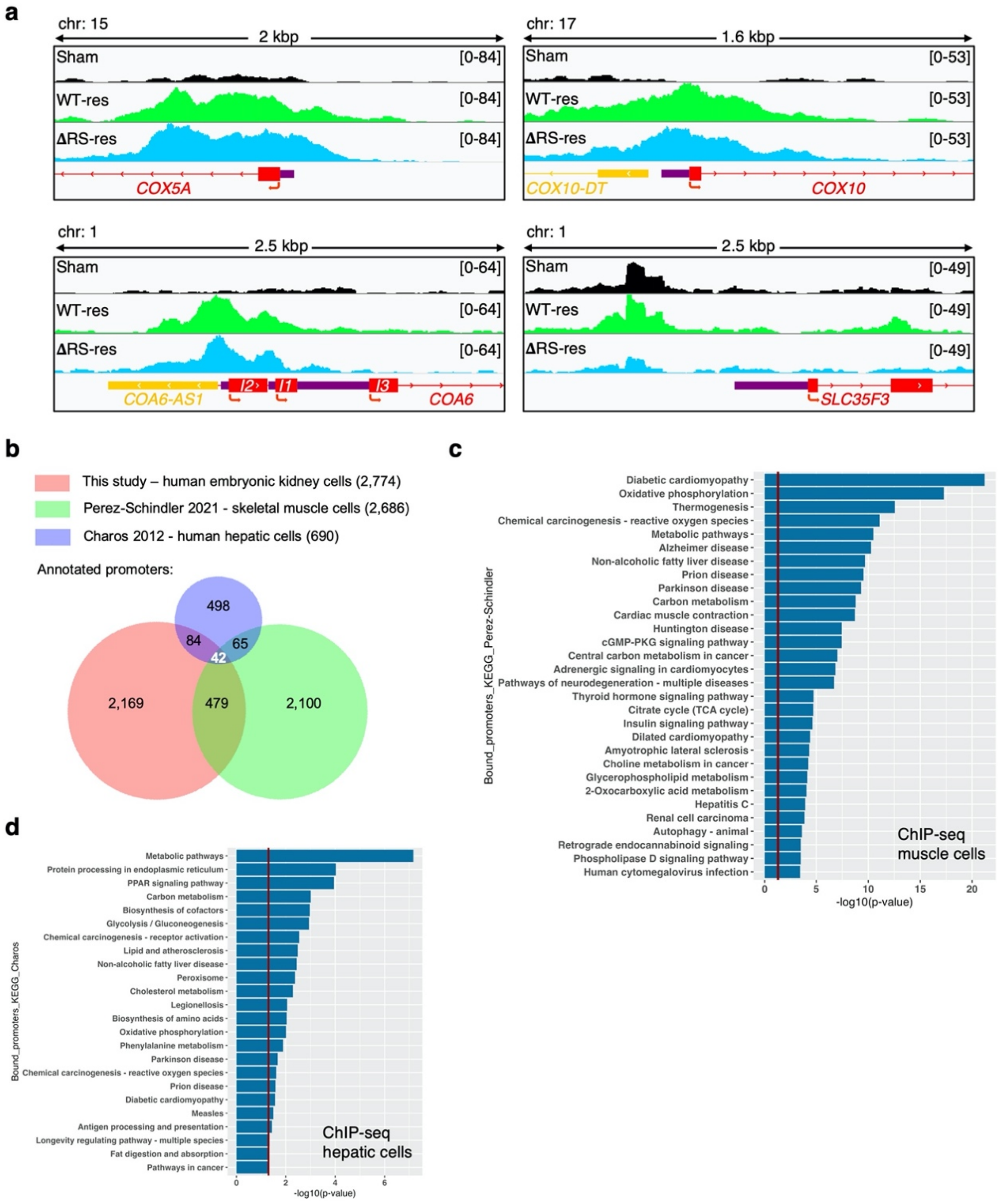

Supplementary Fig. 9 | Genome-wide analysis of PGC-1α-binding to promoters. (**a**) Integrative Genomics Viewer (IGV) snapshot of representative ChIP-seq peaks at gene promoters. *COX5A, COX10* and *COA6* are typical PGC-1α promoter targets involved in OXPHOS and thermogenesis, while *SLC35F3* is directly upstream of *COA6* and is not bound by PGC-1α serving as a negative control. Labeled in red: exons and introns; purple: 5'UTR; yellow: divergent transcript or antisense RNA gene; I1, 2, 3: isoform 1, 2, 3 start site. The sizes of the genome represented in the ChIP-seq traces are indicated between arrows in kbp. (**b**) Venn diagram comparing PGC-1α ChIP-seq in HEK cells (this study), mouse skeletal muscle (Perez-Schindler *et al.*, 2021) and human hepatic cells (Charos *et al.*, 2012). (**c-d**) KEGG pathway analysis for ChIP-seq in muscle cells (c) and ChIP-seq in hepatic cells (d). The statistical analysis uses a modified Fisher's Exact test within the DAVID ontology package.

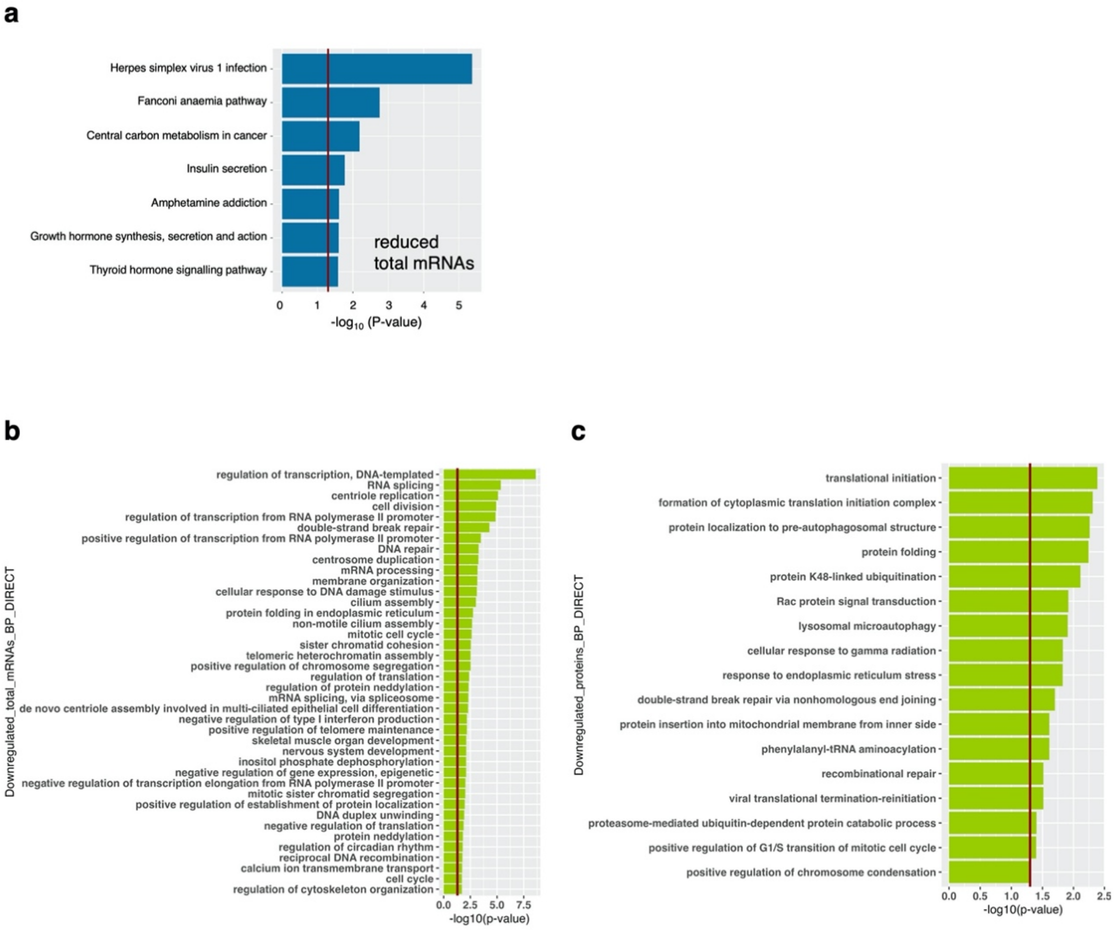

Supplementary Fig. 10 | Genome-wide analysis of PGC-1α-dependent RNA expression. (**a**) KEGG pathway analysis for PGC-1α-dependent reduction of total mRNA expression. (**b-c**) Biological Process (BP direct) bar charts of RS domain mediated down-regulation of total mRNA expression (b) and proteins (c). For all charts, the statistical analysis uses a modified Fisher's Exact test within the DAVID ontology package.

Supplementary Table 1 | Plasmids used and generated in this study.

Plasmid Construct	Vector Backbone	Tag	Cloning Strategy	Reference
PGC-1α 1-798 (FL)	pET24b	N-terminal – GB1 and 6xHis	PCR + NdeI/XhoI	This study
PGC-1α 1-234 (D1)	pET24b	N-terminal – GB1 and 6xHis	PCR + NdeI/XhoI	This study
PGC-1α 254-564 (D2)	pET24b	N-terminal – GB1 and 6xHis	PCR + NdeI/XhoI	This study
PGC-1α 565-798 (D3)	pET24b	N-terminal – GB1 and 6xHis	PCR + NdeI/XhoI	This study
PGC-1α 565-633 (RS)	pET24b	N-terminal – GB1 and 6xHis	PCR + NdeI/XhoI	This study
PGC-1α 634-755 (RRM)	pET24b	N-terminal – GB1 and 6xHis	PCR + NdeI/XhoI	This study
PGC-1α 756-798 (CBM)	pET24b	N-terminal – GB1 and 6xHis	PCR + NdeI/XhoI	This study
PGC-1α 634-798 (RRM+CBM)	pET24b	N-terminal – GB1 and 6xHis	PCR + NdeI/XhoI	This study
PGC-1α 1-798 (FL)	pcDNA5 FRT/TO	N-terminal 3xFLAG	PCR + BamHI/NotI	This study
Control RNAi	pcDNA6.2-GW/EmGFP	miRNA cloned in 3'-UTR EmGFP	BLOCK-iT Kit + PCR + HindIII/NotI	[10]
PGC-1α RNAi (targeting D1)	pcDNA6.2-GW/EmGFP	miRNA cloned in 3'-UTR EmGFP	BLOCK-iT Kit	This study
PGC-1α RNAi (targeting D2)	pcDNA6.2-GW/EmGFP	miRNA cloned in 3'-UTR EmGFP	BLOCK-iT Kit	This study
PGC-1α RNAi (targeting D1 and D2)	pcDNA6.2-GW/EmGFP	miRNA cloned in 3'-UTR EmGFP	D2 excised by BamHI/XhoI + D1 linearized by BglII/XhoI	This study
Control RNAi	pcDNA5/FRT/TO	miRNA cloned in 3'-UTR EmGFP	BLOCK-iT Kit + PCR + HindIII/NotI	[10]
PGC-1α RNAi (targeting D1 and D2)	pcDNA5/FRT/TO	miRNA cloned in 3'-UTR EmGFP	BLOCK-iT Kit + PCR + HindIII/NotI	This study
PGC-1α WT res	pcDNA5/FRT/TO	N-terminal 3xFLAG	BamHI/NotI	This study
PGC-1α ΔRS res	pcDNA5/FRT/TO	N-terminal 3xFLAG	Inverse PCR of WT-res	This study
NXF1/TAP	pGEX4T	N-terminal GST	PCR + BamHI/XhoI	[11]
NXF1/TAP	pIRES-Neo	C-terminal 13myc	PCR+ BamHI/XhoI	[12]
p15/NXT1	pET9a	none	PCR + NdeI/BamHI	[12]

MAGOH	pET24b	C-terminal 6xHis	PCR + NdeI/XhoI	[13]
Aly/REF	p3xFLAG	N-terminal 3xFLAG	PCR + EcoRI/XbaI	[12]
E1B-AP5/ hnRNPUL1	p3xFLAG	N-terminal 3xFLAG	PCR + HindIII/XbaI	[11]
GFP	p3xFLAG	N-terminal 3xFLAG	PCR + EcoRI/XbaI	[13]

Supplementary Table 2 | Antibodies used in this study.

**Antibody**	**Host species**	**Dilution**	**Supplier**
6xHIS	Mouse	1:2000 WB	Proteintech 66005-1
ALYREF	Mouse	1:2000 WB	Sigma A9979, clone 11G5
Class III Beta Tubulin	Chicken	1:1000 WB	Millipore AB9354
c-Myc	Mouse	1:1000 WB	Thermo Fisher MA1-980
FLAG	Mouse	1:2000 WB, 1:1000 IF	Sigma F1804, clone M2
GLS	Rabbit	1:1000 WB	Abcam ab156876
NHEJ1	Rabbit	1:2000 WB	Proteintech 11888-1-AP
NXF1	Rabbit	1:200 IF	Abcam ab129160
NXF1	Mouse	1:2000 WB	Abcam ab50609
PGC-1α	Mouse	1:1000 WB/ 1:1000 IF/ 1:1000 PLA	Calbiochem ST1202
NUP107 (ab1)	Rabbit	1:1000 PLA	Proteintech 19217-1-AP
NUP107 (ab2)	Rabbit	1:1000 PLA	Invitrogen PA5-30774
SSRP1	Mouse	1:1000 WB	Abcam ab26212
TFAM (C-9)	Mouse	1:1000 WB	Santa Cruz sc-376672
Total OXPHOS WB cocktail	Mouse	1:1000 WB	Abcam ab110411
α-tubulin	Mouse	1:10,000 WB	Sigma, clone DM1A
XRCC5	Rabbit	1:2500 WB	Proteintech 16389-1-AP
XRCC6	Rabbit	1:2500 WB	Proteintech 10723-1-1AP
HRP-conjugated chicken	Rabbit	1:5000 WB	Promega G1351
HRP-conjugated mouse	Goat	1:5000 WB	Promega W4011
HRP-conjugated rabbit	Goat	1:5000 WB	Promega W4021
Mouse AlexaFluor 647	Donkey	1:500 IF	Jackson ImmunoResearch 715-605-151
Rabbit AlexaFluor 488	Donkey	1:500 IF	Jackson ImmunoResearch 711-545-152

Supplementary Table 3 | Sequences of qPCR primers used in this study.

Primer	Sequence	Reference
Human *B2M* Forward	5'-CCAGCAGAGAATGGAAAGTCAA-3'	[[14]]
Human *B2M* Reverse	5'-TCTCTCTCCATTCTTCAGTAAGTCAACT-3'	[[14]]
Human *CAT* Forward	5'-ACCAAGGTTTGGCCTCACAA-3'	Origene
Human *CAT* Reverse	5'-GCGGTTAGTGTCAGGATAGG-3'	Origene
Human *COX5A* Forward	5'-CAGATGAGGAGTTTGATGCTCGC-3'	Origene
Human *COX5A* Reverse	5'-GCAGCATCAATGATTTTGGGCTC-3'	Origene
Human *COX10* Forward	5'-TCCTGTGCTGCCAACTCCATCA-3'	Origene
Human *COX10* Reverse	5'-CCAAGGTCAGAATGGCAACTCC-3'	Origene
Human *DDX19B* Forward	5'- GCTTCTCCAAGGAGTCTATGCC-3'	Origene
Human *DDX19B* Reverse	5'- CAGTACCAGACTGAGATTGGGC-3'	Origene
Human *GLS* Forward	5'- CAGAAGGCACAGACATGGTTGG-3'	Origene
Human *GLS* Reverse	5'- GGCAGAAACCACCATTAGCCAG-3'	Origene
Human *KPNA2* Forward	5'- CTGTTGGCTCTCCTTGCAGTTC-3'	Origene
Human *KPNA*2 Reverse	5'- GCAGGATTCTTGTTGCGGCAAAG-3'	Origene
Human *MT-ND1* Forward	5'-CCCTAAAACCCGCCACATCT-3'	[[15]]
Human *MT-ND1* Reverse	5'-GAGCGATGGTGAGAGCTAAGGT-3'	[[15]]
Human *NHEJ1* Forward	5'-CAGTCACCACACAAGAGGTCCA-3'	Origene
Human *NHEJ1* Reverse	5'-TGAGGAGACCAGTTGTTCTGGC-3'	Origene
Human *NRF1* Forward	5'-TTCTCCCGAGGACACCTCTT-3'	Origene
Human *NRF1* Reverse	5'-GGCCGTTTCCGTTTCTTTCC-3'	Origene
Human *NUP58* Forward	5'- GCTTTAGCGGCACAACTTCAGTC-3'	Origene
Human *NUP58* Reverse	5'- TGGCTTCTGCTCGCCTTGTTTC-3'	Origene
Human *NUP62* Forward	5'- CCTTCCAACCAGCCACAAGTAC-3'	Origene
Human *NUP62* Reverse	5'- CGAAGCAAGAGTCGCTGTTCCA-3'	Origene
Human *NUP98* Forward	5'- GGAACCTGTGTCTGCCTCAACA-3'	Origene
Human *NUP98* Reverse	5'- CTTTGGAAGGCAGGCGACTGAA-3'	Origene
Human PGC1α CDS Forward	5'-GTGAAGACCAGCCTCTTTGC-3'	Primer-BLAST (NCBI)
Human PGC-1α CDS Reverse	5'-TCACGTCTCCATCTGTCAGC-3'	Primer-BLAST (NCBI)
Human PGC-1α 3'UTR Forward	5'-GTGTGGAGTCCCTGGAATGG-3'	Primer-BLAST (NCBI)
Human PGC-1α 3'UTR Reverse	5'-GTGTTGGGCGAGAGAAAGGA-3'	Primer-BLAST (NCBI)
Human *TFAM* Forward	5'-AAGATTCCAAGCTAAGGGTGA-3'	Origene
Human *TFAM* Reverse	5'-AGAAGATCCTTTCGTCCAACTTCA-3'	Origene
Human U1 snRNA Forward	5'-CCATGATCACGAAGGTGGTT-3'	Primer-BLAST (NCBI)
Human U1 snRNA Reverse	5'-ATGCAGTCGAGTTTCCCACA-3'	Primer-BLAST (NCBI)
Human *XPO1* Forward	5'- CTACATCTGCCTCTCCGTTGCT-3'	Origene
Human *XPO1* Reverse	5'- CCAATACTTCCTCTGGTTTAGCC-3'	Origene
Human *XRCC*5 Forward	5'-GTTCTAAAGGTCTTTGCAGCAAGA-3'	Origene
Human *XRCC5* Reverse	5'-AAAAGCCACGCCGACTTGAGGA-3'	Origene
Human *XRCC*6 Forward	5'-GGTTTCAACCCGTTGGTACTGC-3'	Origene
Human *XRCC6* Reverse	5'-CTGCAGACACTTGATGAGCAGAG-3'	Origene

Supplementary Note 1 | DNA oligonucleotides used to build the PGC-1α miRNAs and confer resistance to RNAi.

For pcDNA6.2-GW/EmGFP-miR PGC-1α Domain 1:
PGC1a miR D1(133) top:
TGCTGAAAGCTGTCTGTATCCAAGTCGTTTTGGCCACTGACTGACGACTTGGACA GACAGCTTT
PGC1a miR D1(133) bottom:
CCTGAAAGCTGTCTGTCCAAGTCGTCAGTCAGTGGCCAAAACGACTTGGATACAG ACAGCTTTC

For pcDNA6.2-GW/EmGFP-miR PGC-1α Domain 2:
PGC1a miR D2(997) top:
TGCTGTGTACCAGAAGACTCACTGTAGTTTTGGCCACTGACTGACTACAGTGACTT CTGGTACA
PGC1a miR D2(997) bottom:
CCTGTGTACCAGAAGTCACTGTAGTCAGTCAGTGGCCAAAACTACAGTGAGTCTT CTGGTACAC

Quick change oligonucleotides used to confer resistance to chained PGC-1α miRNAs (while not altering the amino-acid sequence of the PGC-1α transgene). Silent mutations are labelled in red:
PGC miR1 res_fwd:
A CTA GAT GTG AAC GAC TTG GAC ACC GAT TCT TTC CTG GGT GGA CTC AAG TGG TGC
PGC miR1 res_rev:
GCA CCA CTT GAG TCC ACC CAG GAA AGA ATC GGT GTC CAA GTC GTT CAC ATC TAG T
PGC miR2 res_fwd:
A AAG AAG CCC AGG TAC AGT GAA AGC AGC GGC ACC CAA GGC AAT AAC TCC ACC AAG
PGC miR2 res_rev:
CTT GGT GGA GTT ATT GCC TTG GGT GCC GCT GCT TTC ACT GTA CCT GGG CTT CTT T

Supplementary References

1. Sullivan AE, Santos SDM. An Optimized Protocol for ChIP-Seq from Human Embryonic Stem Cell Cultures. *STAR Protoc* **1**, 100062 (2020).

2. Cheng J*, et al.* Transcriptional maps of 10 human chromosomes at 5-nucleotide resolution. *Science (New York, NY)* **308**, 1149-1154 (2005).

3. Kapranov P*, et al.* RNA maps reveal new RNA classes and a possible function for pervasive transcription. *Science (New York, NY)* **316**, 1484-1488 (2007).

4. Love MI, Huber W, Anders S. Moderated estimation of fold change and dispersion for RNA-seq data with DESeq2. *Genome Biol* **15**, 550 (2014).

5. Sterne-Weiler T, Weatheritt RJ, Best AJ, Ha KCH, Blencowe BJ. Efficient and Accurate Quantitative Profiling of Alternative Splicing Patterns of Any Complexity on a Laptop. *Mol Cell* **72**, 187-200 e186 (2018).

6. Jiang X*, et al.* Sensitive and Accurate Quantitation of Phosphopeptides Using TMT Isobaric Labeling Technique. *Journal of Proteome Research* **16**, 4244-4252 (2017).

7. Gygi JP, Yu Q, Navarrete-Perea J, Rad R, Gygi SP, Paulo JA. Web-Based Search Tool for Visualizing Instrument Performance Using the Triple Knockout (TKO) Proteome Standard. *Journal of Proteome Research* **18**, 687-693 (2019).

8. Storey AJ*, et al.* ProteoViz: a tool for the analysis and interactive visualization of phosphoproteomics data. *Molecular omics* **16**, 316-326 (2020).

9. Maes E, Hadiwikarta WW, Mertens I, Baggerman G, Hooyberghs J, Valkenborg D. CONSTANd : A Normalization Method for Isobaric Labeled Spectra by Constrained Optimization. *Molecular & Cellular Proteomics* **15**, 2779-2790 (2016).

10. Hautbergue GM*, et al.* UIF, a New mRNA export adaptor that works together with REF/ALY, requires FACT for recruitment to mRNA. *Current Biology* **19**, 1918-1924 (2009).

11. Williams BJL*, et al.* The prototype gamma-2 herpesvirus nucleocytoplasmic shuttling protein, ORF 57, transports viral RNA through the cellular mRNA export pathway. *The Biochemical journal* **387**, 295-308 (2005).

12. Hautbergue GM, Hung M-L, Golovanov AP, Lian L-Y, Wilson SA. Mutually exclusive interactions drive handover of mRNA from export adaptors to TAP. *Proceedings of the National Academy of Sciences of the United States of America* **105**, 5154-5159 (2008).

13. Cruz-Migoni A*, et al.* A Burkholderia pseudomallei toxin inhibits helicase activity of translation factor eIF4A. *Science (New York, NY)* **334**, 821-824 (2011).

14. Grady JP*, et al.* Accurate measurement of mitochondrial DNA deletion level and copy number differences in human skeletal muscle. *PloS one* **9**, e114462 (2014).

15. Campbell GR*, et al.* Mitochondrial DNA deletions and depletion within paraspinal muscles. *Neuropathology and applied neurobiology* **39**, 377-389 (2013).
