# Supplementary material for: The master energy homeostasis regulator PGC-1α exhibits an mRNA nuclear export function
Source: Nat Commun. 2023 Sep 7;14:5496. doi: 10.1038/s41467-023-41304-8 (PMC10485026; doi:10.1038/s41467-023-41304-8)
Supplement: Supplementary file 3 — Description of Additional Supplementary Files [file 41467_2023_41304_MOESM3_ESM.pdf]

Description of Additional Supplementary Files

File Name: Supplementary Data 1
Description: **ChIP-seq counts and promoter identification.** This Excel file contains 5 tabs. (**Tabs 1-3**) Chromatin immunoprecipitation-next generation sequencing (ChIP-seq) for Sham, PGC-1α WT-res and ΔRS-res cell lines induced with doxycycline for 6 days. 1. ChIP-seq; 2. PGC1α-bound promoters; 3. PGC1α-ΔRS-bound promoters; (**Tab 4**) 4. ChIP Pérez-Schindler prom.; (**Tab 5**) 5. ChIP-seq Charos prom.

File Name: Supplementary Data 2
Description: **RNA-seq statistics.** Illumina RNA-sequencing statistics for PGC-1α WT-res and ΔRS-res cell lines induced with doxycycline for 6 days. Data are presented for Whole Cell Transcriptomes (WCT) and Cytoplasmic Transcriptomes (CyT).

File Name: Supplementary Data 3
Description: **RNA-seq counts and transcriptome coverage.** Data are presented for Whole Cell Transcriptomes (WCT) and Cytoplasmic Transcriptomes (CyT) from PGC-1α WT-res and ΔRS-res cell lines induced with doxycycline for 6 days. This Excel file contains 2 tabs: 1. WCT RNA-seq; 2. CyT RNA-seq.

File Name: Supplementary Data 4
Description: **Differential RNA-seq expression analysis.** RNA changes are presented for Whole Cell Transcriptomes (WCT) and Cytoplasmic Transcriptomes (CyT) from PGC-1α WT-res and ΔRS-res cell lines induced with doxycycline for 6 days. This Excel file contains 4 tabs: 1. WCT; 2. CyT; 3. PGC1α RNA NE targets; 4. Alternative splicing.

File Name: Supplementary Data 5
Description: **TMT Mass spectrometry.** Protein changes are presented from PGC-1α WT-res and ΔRS-res cell lines induced with doxycycline for 6 days.

File Name: Supplementary Data 6
Description: **Gene ontology analysis.** Data are based on DAVID Fuctional Annotation Clustering based on GOTERM BP-FAT, CC-FAT, MF_FAT and KEGG pathways (recommended medium stringency, April 2023). This Excel file contains 5 tabs: 1. Reduced proteins; 2. Reduced mRNA expression; 3. PGC1α-bound promoters; 4. PGC1α mRNA NE targets; 5. Alternative splicing.
