# Supplementary material for: The master energy homeostasis regulator PGC-1α exhibits an mRNA nuclear export function
Source: Nat Commun. 2023 Sep 7;14:5496. doi: 10.1038/s41467-023-41304-8 (PMC10485026; doi:10.1038/s41467-023-41304-8)
Supplement: Supplementary file 10 — Reporting Summary [file 41467_2023_41304_MOESM10_ESM.pdf]

nature portfolio

Corresponding author(s): Guillaume Hautbergue

Last updated by author(s): Jul 12, 2023

Reporting Summary

Nature Portfolio wishes to improve the reproducibility of the work that we publish. This form provides structure for consistency and transparency in reporting. For further information on Nature Portfolio policies, see our Editorial Policies and the Editorial Policy Checklist.

Statistics

For all statistical analyses, confirm that the following items are present in the figure legend, table legend, main text, or Methods section.

n/a	Confirmed	
☐	☒	The exact sample size (*n*) for each experimental group/condition, given as a discrete number and unit of measurement
☐	☒	A statement on whether measurements were taken from distinct samples or whether the same sample was measured repeatedly
☐	☒	The statistical test(s) used AND whether they are one- or two-sided<br>*Only common tests should be described solely by name; describe more complex techniques in the Methods section.*
☒	☐	A description of all covariates tested
☐	☒	A description of any assumptions or corrections, such as tests of normality and adjustment for multiple comparisons
☐	☒	A full description of the statistical parameters including central tendency (e.g. means) or other basic estimates (e.g. regression coefficient) AND variation (e.g. standard deviation) or associated estimates of uncertainty (e.g. confidence intervals)
☒	☐	For null hypothesis testing, the test statistic (e.g. *F*, *t*, *r*) with confidence intervals, effect sizes, degrees of freedom and *P* value noted<br>*Give P values as exact values whenever suitable.*
☒	☐	For Bayesian analysis, information on the choice of priors and Markov chain Monte Carlo settings
☒	☐	For hierarchical and complex designs, identification of the appropriate level for tests and full reporting of outcomes
☒	☐	Estimates of effect sizes (e.g. Cohen's *d*, Pearson's *r*), indicating how they were calculated

Our web collection on statistics for biologists contains articles on many of the points above.

Software and code

Policy information about availability of computer code

Data collection	Protocols and softwares used for the analysis of ChIP-seq, RNA-seq and TMT mass spectrometry are all described in Supplementary information.
Data analysis	Raw files were processed using MaxQuant v1.6.2.10. "proteinGroups.txt" file was analyzed using R code version 3.6.3 and RStudio version 1.2.5033. Data is provided in 6 Supplementary Tables (1-6). Excel was used to filter lists of differentially expressed DNA or RNA. Venn diagram was generated using VennDiagram package v1.6.20. Codes are available on request.

For manuscripts utilizing custom algorithms or software that are central to the research but not yet described in published literature, software must be made available to editors and reviewers. We strongly encourage code deposition in a community repository (e.g. GitHub). See the Nature Portfolio guidelines for submitting code & software for further information.

Data

Policy information about availability of data

All manuscripts must include a data availability statement. This statement should provide the following information, where applicable:

- Accession codes, unique identifiers, or web links for publicly available datasets
- A description of any restrictions on data availability
- For clinical datasets or third party data, please ensure that the statement adheres to our policy

All data associated with this study are presented in the main text, supplementary information and the source data file. The ChIP-seq and RNA-seq data have been deposited in Gene Expression Omnibus (GEO) under accession number GSE230429. The TMT mass spectrometry data have been deposited to the ProteomeXchange Consortium (http://proteomecentral.proteomexchange.org) via the PRIDE partner repository (doi: 10.1093/nar/gkab1038) with the dataset identifier PXD031189.

March 2021

Field-specific reporting

Please select the one below that is the best fit for your research. If you are not sure, read the appropriate sections before making your selection.

☒ Life sciences ☐ Behavioural & social sciences ☐ Ecological, evolutionary & environmental sciences

For a reference copy of the document with all sections, see nature.com/documents/nr-reporting-summary-flat.pdf

Life sciences study design

All studies must disclose on these points even when the disclosure is negative.

Sample size	- For stable HEK FlpIN cells, we have used this cell line for multiple experiments. Experiments were repeated in independent biologiocal triplicates.<br>- For HEK 293 cells, we have used this cell line for experiments where plasmids have been transfected. Experiments were repeated in independent biological triplicates.
Data exclusions	We have not excluded any data points from our analysis.
Replication	Experiments have been performed at least in independent biological triplicates. The number of replicates is indicated in figure legends.
Randomization	No randomization was used.
Blinding	Most experiments have been performed by several researchers and the results were only collated and analysed globally once all data were collected.

Reporting for specific materials, systems and methods

We require information from authors about some types of materials, experimental systems and methods used in many studies. Here, indicate whether each material, system or method listed is relevant to your study. If you are not sure if a list item applies to your research, read the appropriate section before selecting a response.

Materials & experimental systems

n/a	Involved in the study
☐	☒ Antibodies
☐	☒ Eukaryotic cell lines
☒	☐ Palaeontology and archaeology
☒	☐ Animals and other organisms
☒	☐ Human research participants
☒	☐ Clinical data
☒	☐ Dual use research of concern

Methods

n/a	Involved in the study
☐	☒ ChIP-seq
☒	☐ Flow cytometry
☒	☐ MRI-based neuroimaging

Antibodies

Antibodies used	All antibodies used in this study are provided in supplementary table 2.
Validation	Depletion and overexpression experiments based on our data and/or the literature. All antibody concentrations were optimised prior to collect data. Information related to antibodies (sources, clone numbers, dilutions, secondary antibodies for detection) is provided in supplementary table 2.

Eukaryotic cell lines

Policy information about cell lines

Cell line source(s)	Stable HEK FLpIN cell lines were generated within the Hautbergue lab according to the Invitrogen manufacturer. HEK-293T were bought from ATCC.
Authentication	None of the cell lines used have been authenticated
Mycoplasma contamination	Cell lines are checked for mycoplasma contamination on a monthly basis. Any contaminated cell lines is discarded and new vials are thawed. Data is only acquired and published using non-contaminated cells.
Commonly misidentified lines<br>(See ICLAC register)	none

ChIP-seq

Data deposition

☒ Confirm that both raw and final processed data have been deposited in a public database such as GEO.

☒ Confirm that you have deposited or provided access to graph files (e.g. BED files) for the called peaks.

Data access links
May remain private before publication.

https://www.ncbi.nlm.nih.gov/geo/query/acc.cgi?acc=GSE230429
The following secure token has been created to allow review of record GSE230429 while it remains in private status:
qritqsauhjknlin

Files in database submission

GSM7221662_Sham_INPUT_R1.bigWig
GSM7221663_PGC1a_WT_INPUT_R1.bigWig
GSM7221664_PGC1a_deltaRS_INPUT_R1.bigWig
GSM7221665_Sham_INPUT_R2.bigWig
GSM7221666_PGC1a_WT_INPUT_R2.bigWig
GSM7221667_PGC1a_deltaRS_INPUT_R2.bigWig
GSM7221668_Sham_INPUT_R3.bigWig
GSM7221669_PGC1a_WT_INPUT_R3.bigWig
GSM7221670_PGC1a_deltaRS_INPUT_R3.bigWig
GSM7221671_Sham_ChIP_R1.bigWig
GSM7221671_Sham_ChIP_R1_peaks.narrowPeak.gz
GSM7221672_PGC1a_WT_ChIP_R1.bigWig
GSM7221672_PGC1a_WT_ChIP_R1_peaks.narrowPeak.gz
GSM7221673_PGC1a_deltaRS_ChIP_R1.bigWig
GSM7221673_PGC1a_deltaRS_ChIP_R1_peaks.narrowPeak.gz
GSM7221674_Sham_ChIP_R2.bigWig
GSM7221674_Sham_ChIP_R2_peaks.narrowPeak.gz
GSM7221675_PGC1a_WT_ChIP_R2.bigWig
GSM7221675_PGC1a_WT_ChIP_R2_peaks.narrowPeak.gz
GSM7221676_PGC1a_deltaRS_ChIP_R2.bigWig
GSM7221676_PGC1a_deltaRS_ChIP_R2_peaks.narrowPeak.gz
GSM7221677_Sham_ChIP_R3.bigWig
GSM7221677_Sham_ChIP_R3_peaks.narrowPeak.gz
GSM7221678_PGC1a_WT_ChIP_R3.bigWig
GSM7221678_PGC1a_WT_ChIP_R3_peaks.narrowPeak.gz
GSM7221679_PGC1a_deltaRS_ChIP_R3.bigWig
GSM7221679_PGC1a_deltaRS_ChIP_R3_peaks.narrowPeak.gz

Genome browser session
(e.g. UCSC)

IGV browser session.

Methodology

Replicates

3 biological replicates

Sequencing depth

100bp, single-end
From FastQC (Trimmed)
Category Unique Reads Duplicate Reads
PGC1a_WT_ChIP_R1_T1_1 42266866 17235443
PGC1a_WT_ChIP_R1_T1_2 43068065 16434244
PGC1a_WT_ChIP_R2_T1_1 36777486 10367865
PGC1a_WT_ChIP_R2_T1_2 37075928 10069423
PGC1a_WT_ChIP_R3_T1_1 32421142 9053974
PGC1a_WT_ChIP_R3_T1_2 32848290 8626826
PGC1a_WT_INPUT_R1_T1_1 43030468 10018830
PGC1a_WT_INPUT_R1_T1_2 43367378 9681920
PGC1a_WT_INPUT_R2_T1_1 38120511 7099482
PGC1a_WT_INPUT_R2_T1_2 38458705 6761288
PGC1a_WT_INPUT_R3_T1_1 32042699 5465656
PGC1a_WT_INPUT_R3_T1_2 32227077 5281278
PGC1a_deltaRS_ChIP_R1_T1_1 34411968 9260138
PGC1a_deltaRS_ChIP_R1_T1_2 34784050 8888056
PGC1a_deltaRS_ChIP_R2_T1_1 36863320 10256705
PGC1a_deltaRS_ChIP_R2_T1_2 37366350 9753675
PGC1a_deltaRS_ChIP_R3_T1_1 37871535 10649482
PGC1a_deltaRS_ChIP_R3_T1_2 38437076 10083941
PGC1a_deltaRS_INPUT_R1_T1_1 44826647 9231189
PGC1a_deltaRS_INPUT_R1_T1_2 45097996 8959840
PGC1a_deltaRS_INPUT_R2_T1_1 41776577 9358436
PGC1a_deltaRS_INPUT_R2_T1_2 42114688 9020325
PGC1a_deltaRS_INPUT_R3_T1_1 34774546 5936832
PGC1a_deltaRS_INPUT_R3_T1_2 34919260 5792118

Sham_ChIP_R1_T1_1 32785724 9109780
Sham_ChIP_R1_T1_2 33307623 8587881
Sham_ChIP_R2_T1_1 33398584 8598594
Sham_ChIP_R2_T1_2 33629097 8368081
Sham_ChIP_R3_T1_1 30649849 8275646
Sham_ChIP_R3_T1_2 31101241 7824254
Sham_INPUT_R1_T1_1 39251655 7546337
Sham_INPUT_R1_T1_2 39604720 7193272
Sham_INPUT_R2_T1_1 37601811 6945151
Sham_INPUT_R2_T1_2 37933271 6613691
Sham_INPUT_R3_T1_1 39516809 7916224
Sham_INPUT_R3_T1_2 39765762 7667271

Antibodies

Specificity was validated by RNAi in our study and other studies cited in the main manuscript (line 251). Anti-FLAG M2 mouse monoclonal antibody (Sigma, F1804)

Peak calling parameters

nextflow run nf-core/chipseq --narrow_peak --input design.csv -profile crick --genome GRCh38 --macs_fdr 0.05

Data quality

The pipeline performs extensive QC and version reporting using MultiQC, FastQC, prseq, deepTools, and phantompeakqualtools. The FDR threshold for peak calling was set at 0.05 (5%). DNA binding was regarded as significant when PGC-1α or PGC-1α-ΔRS versus Sham was log2FC >0.5 and p-value <0.05

Software

For sequence analysis, the nf-core/chipseq, written in the Nextflow domain-specific language (ver. 22.04.0), was used to perform the primary analysis of the samples in conjunction with Singularity (ver. 3.6.4). The command used was "nextflow run nf-core/chipseq --narrow_peak --input design.csv -profile crick --genome GRCh38 --macs_fdr 0.05".
